# Interplay of ELF5, GATA-3 and FLI-1 regulate malignant transformation of keratinocytes

**DOI:** 10.1038/s41598-026-59939-0

**Published:** 2026-07-16

**Authors:** Hetal. J. Patel, Maximilian. E. Pickup, Maryam. A. Lawal, Chandrakala Khadka, Mohammed. I. Ahmed

**Affiliations:** 1 School of Science and Technology, Nottingham Trent University, Nottingham, NG11 8NS UK; 2 Baillie-Gifford Pandemic Science Hub, Institute for Regeneration and Repair, University of Edinburgh, Edinburgh, UK

**Keywords:** ELF5, Keratinocytes, Proliferation, Differentiation, cSCC, Cancer, Cell biology, Molecular biology

## Abstract

Cutaneous squamous cell carcinoma (cSCC) arises from epidermal keratinocytes and is the second most common epithelial malignancy. E74-like factor 5 (ELF5) is a member of the epithelium specific ETS subfamily of transcription factors and is essential for epithelial development, homeostasis and prevention of epithelial tumourigenesis. Transformation of keratinocytes involves the disruption of the progenitor differentiation program causing keratinocytes to fail to complete the process. This can lead to dysplastic epithelium and precancerous cells in skin. However, the precise mechanisms that causes the failure of keratinocytes to differentiate completely is not well understood. In our study, we discovered that ELF5 is abundantly expressed in healthy human skin (both basal and suprabasal layers), while its expression is lost in cSCC tissue biopsies. Using CRISPR-Cas9 knockout technology, functional studies along with transcript and protein analysis, loss of ELF5 in immortalised healthy keratinocytes leads to decreased ability of cells to differentiation and increased migration and tumourigenicity, in vitro. Overexpression of ELF5 leads to inhibition of cell migration and proliferation in keratinocytes and in cancer cell lines (A431 and SCC-9), in vitro. Further analysis using RNA transcriptomics has uncovered that GATA-3 and FLI-1 are potentially key molecular targets of ELF5 in keratinocytes. ELF5 regulation of both GATA-3 and FLI-1 is required to maintain the balance between healthy keratinocytes (proliferation/differentiation) and to inhibit precancerous cell formation (tumourigenicity). Our data indicates that ELF5 plays an important role in potentially preventing transformation of keratinocytes and could suppresses skin tumourigenesis. However, additional studies are required to determine this, which could lead the targeting of ELF5 for future drug treatments in cSCC. Better understanding of the underlying mechanisms by which ELF5 regulates gene expression and consequently cell activity will provide new knowledge that can be translated into long-term benefits for patients with precancerous lesions to cSCC.

**Supplementary Information:**

The online version contains supplementary material available at 10.1038/s41598-026-59939-0.

## Introduction

Cutaneous squamous cell carcinoma (cSCC) arises from epidermal keratinocytes and is the second most common epithelial malignancy^1^, with an annual incidence of more than 1 million in the USA^1^ and over 45,000 incidences every year in England^2^. The global age-standardised prevalence of cSCC was 39.29 per 100 000 population, the highest among the three common skin cancers, including malignant skin melanoma (25.97 per 100 000 population) and basal cell carcinoma (5.15 per 100 000 population)^3,4^. The stepwise progression from healthy epidermal keratinocytes to precancerous lesions and subsequent cSCC involves mutations in pathways that govern cell division, intercommunication, apoptosis and differentiation^5^. Transformation of keratinocytes disrupts the progenitor differentiation program causing keratinocytes in cSCC formation to fail to complete the differentiation process, leading to precancerous cells and dysplastic skin epithelium. However, the precise mechanisms that causes the failure of keratinocytes to differentiate completely is not well understood.

E74-like factor 5 (ELF5) is a member of the E26 transformation specific (ETS) family of transcription factors, which is expressed predominantly in epithelial cells and has been shown to be a critical transcriptional regulator of cell fate in stem/progenitor cells by regulating target genes, such as, BMP and Notch signalling pathways^6–9^, which are involved in cell proliferation and differentiation processes in development of epithelial tissues, including the kidney^8^, mammary gland^10^ and lung^6^. In skin, we have shown that Elf5 is a direct target of miR-148a required for stem/progenitor cell regulation and Elf5 is also required for controlling keratinocyte proliferation and differentiation processes in mice skin^11,12^. In cancer, ELF5 has been shown to control genes involved in cell proliferation, differentiation, apoptosis, and inhibition of metastasis in human cancers^7,13–15^, indicating an important tumour suppressive role in many cancers. However, ELF5 preventative role in cSCC initiation and development is unknown. In this study, we demonstrate that ELF5 shows distinct and elevated expression patterns in healthy skin, with reduced or lost expression in cSCC tissues and cancerous cell lines. Modulation of ELF5 activities can affect keratinocyte proliferation, differentiation, migration and tumourigenicity, in vitro. These effects are accompanied by dramatic changes in the gene expression and activity of different signalling pathways including GATA-3 and FLI-1. Furthermore, we identified GATA-3 and FLI-1 as targets of ELF5, which suggests that ELF5 is an important regulator of cellular processes such as proliferation, differentiation as well as tumourigenesis. Here, we believe we have identified a gap in knowledge in the precise mechanisms that causes the transformation of healthy keratinocytes to potential precancerous/cancerous cell formations, in particular, the failure of keratinocytes to fully differentiate leading to dysplastic epithelium formation and progression to cSCC.

## Results

### ELF5 expression is reduced in cSCC compared to healthy skin tissue

**Fig. 1:**
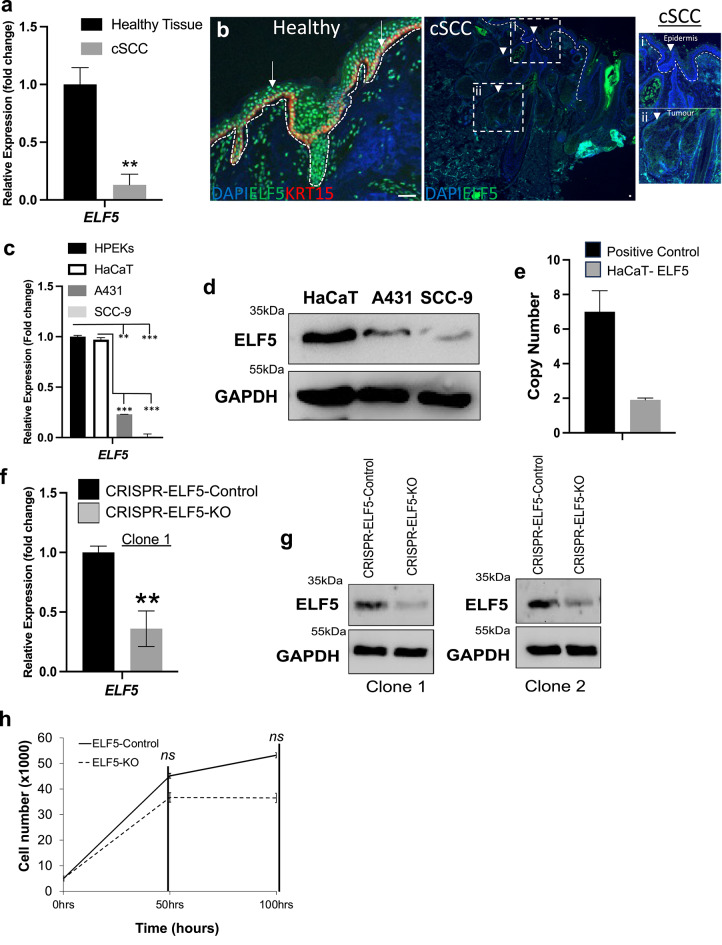
Expressional analysis of ELF5 and CRISPR-Cas9 targeting of *ELF5* in keratinocytes. (**a**) *ELF5* expression is dramatically downregulated in cSSC tissue biopsies compared to healthy skin tissue.* n* = 3 samples per tissue group. Data are presented as mean ± SEM values from three experimental repeats. **p < 0.01, unpaired Student’s *t*-test. SEM ± values (**b**) ELF5 protein expression is also lost in the epidermis and tumour lesions of cSCC biopsies (arrowheads, dotted box inserts) compared to abundant nuclear ELF5 expression observed in the epidermis of healthy tissues (basal ELF5 + /KRT15 + and suprabasal layers, arrows). (**c, d**) RT-qPCR and protein analysis in human primary epidermal keratinocytes (HPEKs), immortalized human keratinocytes (HaCaT) and human squamous carcinoma cell lines (A431 and SCC-9); *ELF5* expression is significantly reduced in A431, and SCC-9 cell lines, compared to healthy keratinocytes (HPEKs and HaCaT cells). (**e**) RT-qPCR analysis of copy number variation determined that there are up to two copies of *ELF5* present in HaCaT cells. Data are presented as mean ± SEM values from three experimental repeats. (**f, g**) This was followed by CRISPR-Cas9 targeting of *ELF5* in HaCaT cells. Several single-cell *ELF5*-KO and control clones were picked and expanded. The correct knockout assembly was confirmed by DNA sequencing (data not shown) and ELF5 expression was assessed using RT-qPCR and Western blots (Panels f and g). We observed significantly reduced expression after loss of ELF5 at both transcript and protein levels compared to controls. Data are presented as mean ± SEM values from three independent repeats. A single representative image of successful CRISPR-Cas9-mediated knockout (KO) of *ELF5* in HaCaT cells for Clones 1 and 2 (Panel g). Clone 1 was then used for all subsequent experiments. (**h**) The effect on cell growth upon CRISPR/Cas9-mediated *ELF5* knockout (ELF5-KO) in HaCaT cells was compared with CRISPR-Cas9 ELF5-Control cells (ELF5-Control) overtime. We observed that loss of *ELF5* (ELF5-KO) does not lead to a significant increase and/or decrease in growth compared to ELF5-Control cells overtime. Data are presented as mean ± SEM values from three experimental repeats. *ns:* not significant, **p < 0.01; ***p < 0.001. unpaired Student’s *t*-test. SEM ± values. DAPI: Blue, ELF5: Green, KRT15: Red. Dashed line demarcates epidermal-dermal boarder. 50 *μ* m scale bar.

Using RT-qPCR, *ELF5* expression was significantly reduced in cSCC tissues samples compared to healthy skin tissue samples (**p value < 0.01, Fig. 1a). Furthermore, ELF5 protein expression was lost in cSCC tissue samples (Fig. 1b, arrowheads and inserts) compared to strong nuclear expression found in the healthy epidermis (basal (Krt15 +) and suprabasal layers) (Fig. 1b, arrows). In addition, we observed downregulation of ELF5 transcript and protein expression in cancerous cell lines (A431 and SCC-9) compared to primary human epidermal keratinocytes (HPEKs) and immortalized human keratinocytes (HaCaT) (Fig. 1c,d). We believe that this data suggests that downregulation of ELF5 may play a role in tumour initiation and progression.

### Loss-or-gain of ELF5 functions in keratinocytes leads to modulation of cell proliferation, differentiation and migration abilities, in vitro

As we observed high ELF5 expression in healthy skin and in immortalized human keratinocytes (HaCaT) (Fig. 1a–d), we next analysed the effects loss-and-gain of ELF5 expression in HaCaT cells by using CRISPR-Cas9-mediated-Knockout (KO) technique and ELF5 overexpression studies. We initially confirmed that HaCaT cell line had up to two copies of *ELF5* allele by CNV assay (Fig. 1e) followed by CRISPR-Cas9-mediated ELF5 KO in HaCaT cell line (Fig. 1f,g). Several single-cell *ELF5*-knockout clones were picked and expanded. The correct knockout assembly was confirmed by DNA sequencing (data not shown), RT-qPCR and protein analysis (**p value < 0.01; Fig. 1f,g). All clones analysed exhibited similar results with respect to ELF5 expression (data shown for two clones, Fig. 1g). Clone 1 was used for subsequent assays and will be referred from now on as ELF5-KO and its corresponding control cell line, ELF5-Control. Cell growth analysis by evaluating the total number of cells; revealed that loss of ELF5 (ELF5-KO) did not significantly impact the ability of cells to grow overtime compared to ELF5-Control cells (Fig. 1h). This is consistent with previous studies where loss of target gene via CRISPR-Cas9 editing did not significantly impact cell growth overtime^16^.

**Fig. 2:**
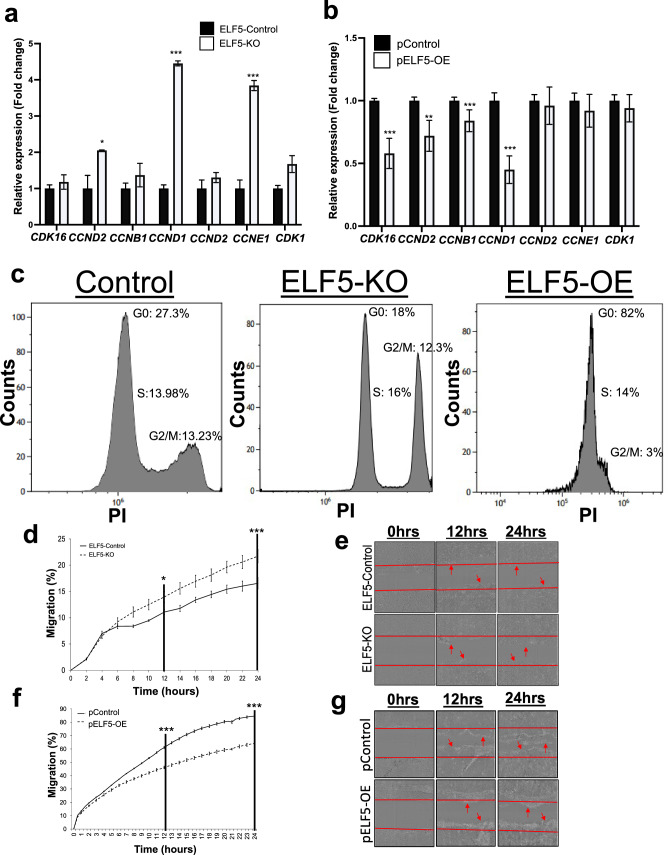
Loss-or-gain of ELF5 function impacts proliferation and migratory abilities of keratinocytes. (**a–g**) RT-qPCR, cell cycle analysis (flow cytometry) and in vitro 2D ‘scratch assay’ analysis using CRISPR/Cas9-mediated *ELF5* knockout (ELF5-KO) and *ELF5* overexpression (pELF5-OE) cells compared to corresponding control cells. (**a–b**) We observed that loss-or-gain of ELF5 expression significantly modulates expression of cell cycle markers. Data are presented as mean ± SEM values from three independent experiments. (**c**) Flow cytometric analysis by PI revealed that loss of ELF5 showed significantly reduced number of cell population at G0 Phase, while cells progressing through the cell cycle were not significantly impacted compared to control cells. While overexpression of ELF5 leads to significant G0-phase arrest of cells and significantly reduced percentage of cells in G2/M compared to control cells. The graphs shown are from a single representative experiment. Percentages are presented as mean values from three independent experiments and statistical analysis can be found in Supplemental Figure S1. (**d–g**) Similarly, using 2D ‘scratch assay’; we observed that loss of ELF5 leads to significantly accelerated migration of keratinocytes, while overexpression of ELF5 inhibits this effect compared to their corresponding controls. Representative images and data presented as *n* = 5 scratches per group. Red line: original wound edge; red arrows: migrating cells. *p < 0.05, ***p < 0.001; unpaired Student’s *t*-test.

Subsequently, we wanted to determine if modulation of ELF5 expression had any impact on cell proliferation and migration. We observed in ELF5-KO cells; a significant increase in expression of proliferation markers, *CCND2, CCND1* and *CCNE1* (*p value < 0.05; ***p value < 0.001, Fig. 2a) was seen compared to ELF5-Control cells. In addition, we transiently overexpressed *ELF5* in HaCaT cells and observed the opposite effects, where expression of *CDK16, CCND2, CCNB1* and *CCND1* were significantly decreased compared to control cells (**p value < 0.01, ***p value < 0.001; Fig. 2b). Furthermore, we performed flow cytometry for cell cycle analysis, and we observed a significant reduction in cells in G0 phase of the cell cycle in ELF5-KO cells (***p value < 0.001) compared to control with no significant impact observed for S and G2/M phases suggesting that cell population is progressing through the cell cycle with limited impact (Fig. 2c and Supplemental Figure S1). In transiently overexpressing ELF5 cells, we observed a significant (***p value < 0.001) cell accumulation in G0-phase and a reduction of cells entering the S and G2/M (***p value < 0.001) phases of the cell cycle compared to ELF5-Controls cells suggesting cell arrest at G0-phase has occurred after overexpression of ELF5 (Fig. 2c and Supplemental Figure S1). We then examined the migratory potential of HaCaT cells after gain-or-loss of ELF5; we observed significant (*p value < 0.05, ***p value < 0.001) accelerated migration in ELF5-KO cells compared to ELF5-Control cells overtime (Fig. 2d,e). Whilst transient overexpression of *ELF5* significantly reduced the ability of keratinocytes to migrate (***p value < 0.001; Fig. 2f,g; Supplemental Figure S2).

**Fig. 3:**
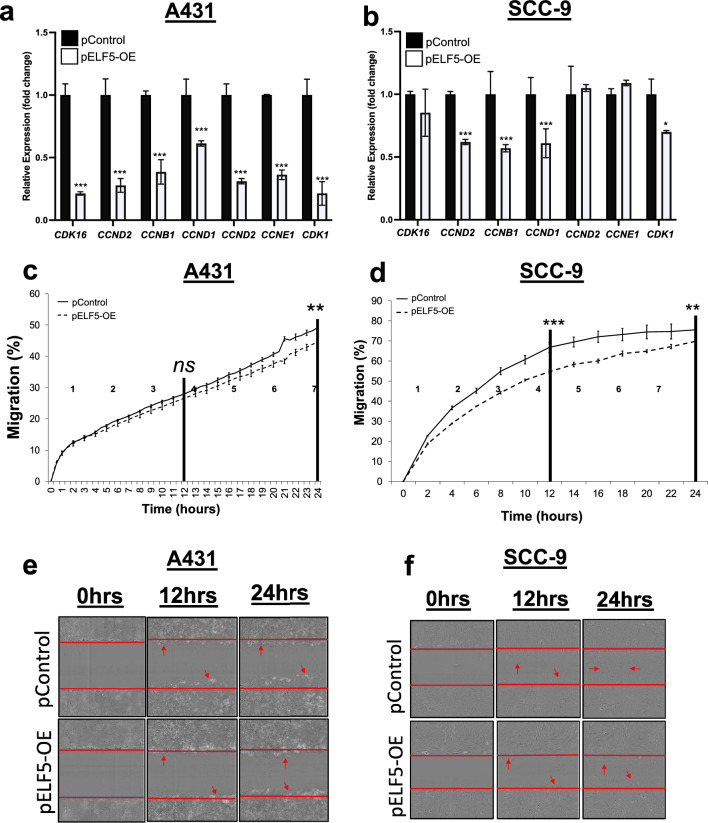
Overexpression of ELF5 inhibits cell proliferation and migration of A431 and SCC-9 cancerous cells lines. (**a, b**) RT-qPCR analysis of cell cycle markers in A431 and SCC-9 cancerous cell lines show a decrease in the expression of majority of genes analysed after overexpression of ELF5 (pELF5-OE) compared to control cells (pControl). Data are presented as mean ± SEM values from three independent experiments. (**c–f**) Using 2D ‘scratch assay’; we observed that overexpression of ELF5 leads to significantly reduced migration of both cancerous cell lines compared to control cells overtime. Representative images and data presented as *n* = 5 scratches per group. Red line: original wound edge; red arrows: migrating cells. ns: not significant, *p < 0.05, ***p < 0.001; unpaired Student’s *t*-test.

Consequently, we assessed the impact of transiently overexpressing *ELF5* in skin cancer cells lines A431 and SCC-9. The A431 cell line (epidermoid, skin-derived cancer cells) is a well-established model for squamous cell carcinoma studies and tumourigenesis and although not derived directly from cutaneous SCC, they share many features with cSCC^17–20^. While SCC-9 represents oral/tongue epithelial cancer biology (head-and-neck) and by comparing the effects of ELF5 expression on these cancer cell lines can highlight whether there is common and broader mechanism involved across squamous epithelial types, which is important for predicting clinical outcomes. After transiently overexpressing *ELF5* in both A431 and SCC-9 cells (Supplemental Figure S2) , we found that it leads to a significant reduction in cell cycle markers *CDK16, CCND2, CCNB1, CCND1, CCND2, CCNE1* and *CDK1* (***p value < 0.001, *p value < 0.05; Fig. 3a,b) for both cell lines. Furthermore, *ELF5* overexpression also leads to a significant inhibition of migration for both cancerous cell lines compared to control cells overtime (**p value < 0.01, ***p value < 0.001, Fig. 3c–f) suggesting that ELF5 is an important regulator of proliferation and migration abilities in these cancer cell lines.

**Fig. 4:**
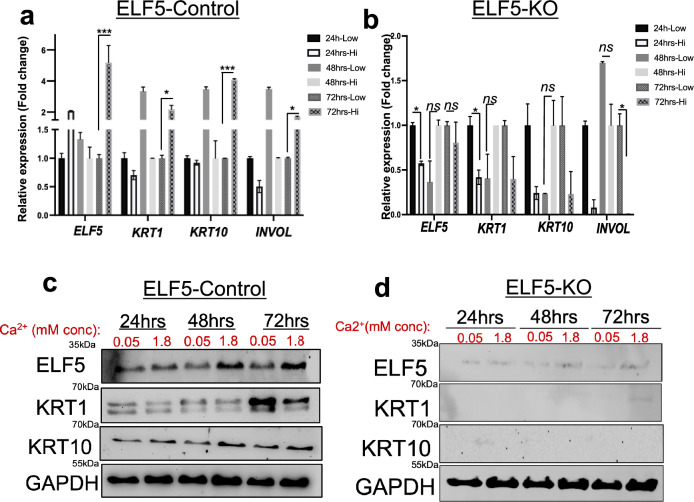
Loss of ELF5 inhibits keratinocytes differentiation process. (**a, b**) RT-qPCR analysis during calcium (Ca2^+^)-induced (low: 0.05 mM or high: 1.8 mM) keratinocyte differentiation in CRISPR/Cas9 HaCaT-Control (ELF5-Control) and CRISPR/Cas9 HaCaT-ELF5 knockout cells (ELF5-KO) cells: ELF5-Control cells were able to differentiation as normal as seen by increased expression of keratinocyte differentiation-associated markers *CYTOKERATIN 1* (*KRT1*), *KRT10,* and *INVOLUCRIN* (*INVOL*) and *ELF5* (panel **a**), respectively. While ELF5-KO cells were unable to differentiate properly (panel **b**). Data are presented as mean ± SEM values from three independent experiments. (**c, d**) Western blot analysis of ELF5, KRT1 and KRT10 during calcium-induced keratinocyte differentiation using CRISPR/Cas9 HaCaT-Control (ELF5-Control) and CRISPR/Cas9 HaCaT-ELF5 knockout cells (ELF5-KO) cells: consistent with RT-qPCR, ELF5, KRT1 and KRT10 protein levels did not increase or were absent during calcium-induced differentiation of ELF5-KO cells (panel d) compared to ELF5-Controls cells (panel c) overtime. Data shown are from a single representative experiment of three experimental repeats. Densiometric analysis is shown in Supplemental Figure S8a-b. *ns:* not significant *p < 0.05, ***p < 0.001; unpaired Student’s *t*-test.

Using calcium-induced keratinocyte differentiation in vitro assay, we assessed the impact of loss of ELF5 on keratinocyte differentiation. We observed that ELF5-KO cells were unable to differentiated properly as observed by reduced transcript expression of differentiation association markers (CYTOKERATIN 1 (*KRT1), KRT10 and INVOL*) and protein expression (KRT1 and KRT10) (Fig. 4b,d) overtime after loss of ELF5 compared with ELF5-controls cells, which were able to differentiate as expected (Fig. 4a,c; Supplemental Figure S8a-b). These data suggests that ELF5 is essential for the maintenance of keratinocyte differentiation processes.

### FLI-1 and GATA-3 are targets of ELF5 in keratinocytes

**Fig. 5:**
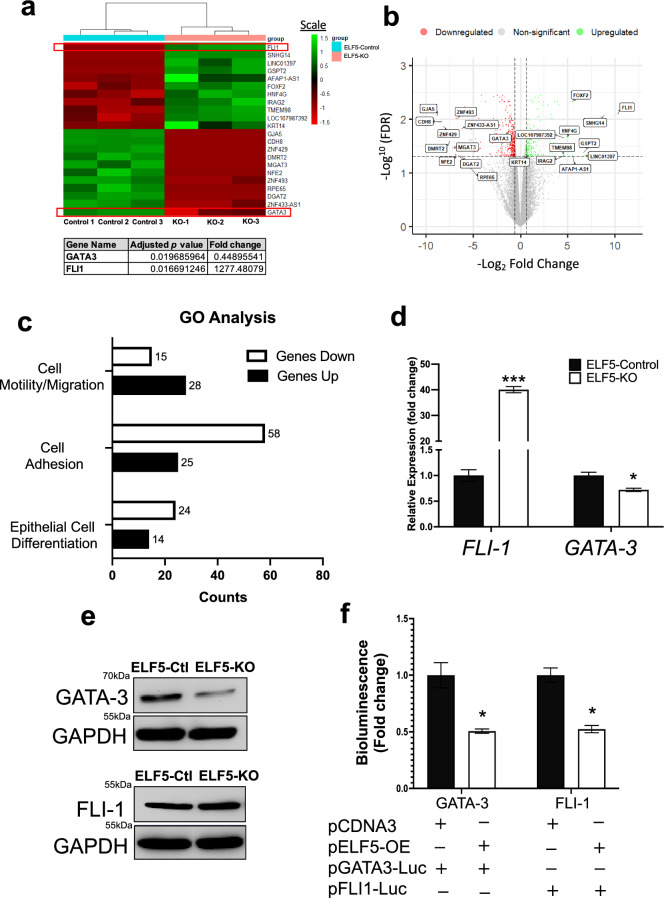
Global gene expression profiling of keratinocytes after CRISPR-Cas9 mediated *ELF5* knockout. (**a**) Heatmap representing significantly increased and/or decreased gene expression values were z-score normalized per gene across all samples. Colours range from − 1.5 to + 1.5 standard deviations from the mean, with red indicating lower expression and green indicating higher expression in CRISPR/Cas9 HaCaT-Control cells (ELF5-Control; Control; ELF5-Ctl) compared to CRISPR/Cas9 HaCaT-ELF5 knockout cells (ELF5-KO; KO). (**b**) Volcano plot of differentially expressed genes between ELF5-KO *versus* ELF5-Control cells. Green coloured point are significantly upregulated genes, red coloured points are significantly downregulated genes (P < 0.05, fold change > 1.5). (**c**) Bar chart depicting the number of genes significantly up-down regulated in Gene Ontology (GO) terms involved in epithelial cell differentiation, cell adhesion and cell motility/migration. (**d**) Validation of RNA-sequencing data; RT-qPCR analysis focusing on gene targets GATA-3 and FLI-1 in ELF5-Control and ELF5-KO cells; loss of ELF5 leads to an increase in the expression of *FLI-1* and decrease in expression for *GATA-*3 compared to ELF5-Control cells, respectively. Data are presented as mean ± SEM values from three independent experiments. (**e**) Western blot analysis: consistent with RT-qPCR, loss of *ELF5* activity leads to an increase and/or a decrease in protein levels of FLI-1 and GATA-3, respectively. Data shown are from a single representative experiment from three independent experiments. Densiometric analysis shown in Supplemental Figure S8c. (**f**) Promoter Reporter assay: HaCaT cells were transfected with plasmids containing human GATA-3 or FLI-1 promoter fragments along with ELF5-overexpressing vector (pELF5-OE) or with pCDNA3 Control vector; decrease in both pGATA-3-Luc or pFLI-1-Luc promoter activities was observed in presence of pELF5-OE plasmid compared to pCDNA3 control plasmid. Data are presented as mean ± SEM values from three experimental repeats; * p < 0.05, ***p < 0.001; unpaired Student’s *t*-test.

To identify potential ELF5 targets in keratinocytes, RNA sequencing followed by differential gene expression analysis was conducted, comparing ELF5-KO cells to ELF5-Control cells (Fig. 5a; Supplemental Figure S3a). We found that 595 genes were significantly differentially expressed with 179 genes being overexpressed and 416 genes being downregulated (FDR < 0.05; Log2FC fold change > 1.5; Fig. 5b). As ELF5 is known to regulate cell differentiation, migration and cell adhesion processes ^9,11–13,16^, we therefore focused our Gene Ontology analysis (Fig. 5c) to the “cell motility/migration, GO: GO:2,000,147 and GO:0,030,335; Q value: 3.77E-05 and 3.37E-05)”, “cell adhesion, GO:0,030,155; Q value: 9.48E-05” and “epithelial cell differentiation, GO:0030855; Q value: 0.006694” categories. Our analysis showed that loss of ELF5 in HaCaT cells leads a greater number of genes being downregulated suggesting epithelial programs are tightly regulated by ELF5 in these cells. This is consistent with previous studies showing that ELF5 is essential for maintaining epithelial identity and promotes differentiation^21^.

Furthermore, loss of ELF5 function has been shown to result in downregulation of adhesion molecules and polarity genes, promoting epithelial-mesenchymal transition (EMT) and increased cellular plasticity^13^. Such downregulation may reflect both impaired epithelial regulation and enhanced susceptibility to invasive or migratory phenotypes, linking ELF5 transcriptional repression to functional changes observed in our HaCaT cells. In addition, a barcode plot for the Gene Ontology term ‘epithelial cell differentiation’ demonstrated that genes associated with this pathway were predominantly downregulated in our differential expression analysis (NES: − 1.338) (Supplemental Figure S3b).

From our RNA sequencing analysis, we focused on FLI-1 and GATA-3 as they were significantly up-and downregulated genes after loss of ELF5 in keratinocytes but are also known crucial regulators of cell growth/survival, differentiation, tumour initiation and progression^22–30^. GATA-3 is expressed in human keratinocytes and is crucial for keratinocyte differentiation and SC lineage determination for a healthy epidermal barrier^31^. Other studies have shown that GATA-3 expression is associated with either survival advantage or disadvantage depending on the cancer type^32^. In contrast, FLI-1 is a widely known oncogene contributing to various malignancies^33^ but has no known role in keratinocytes or cSCC formation. At first, we determined FLI-1 and GATA-3 transcript expression in healthy skin and cSCC samples and found that GATA-3 is significantly reduced in cSCC skin biopsies compared to healthy skin (**p value < 0.01, Supplemental Figure S4a). This is consistent with previous studies where GATA-3 expression is decreased with cutaneous SCC progression^26^.While FLI-1 expression is significantly increased in cSCC skin biopsies compared to healthy tissues at transcript and protein levels (***p value < 0.001, Supplemental Figure S4a,b).

Subsequently, we validated our bioinformatic data and we observed that loss of ELF5 results in reduction in both transcripts and protein expression of GATA-3 (*p value < 0.05, Fig. 5d,e, Supplemental Figure S8c). Additionally, FLI-1 expression is increased at both at transcript and protein levels in our ELF5-KO cells compared to ELF5-Control cells (***p value < 0.001; Fig. 5d,e, Supplemental Figure S8c). Furthermore, transiently overexpressing *ELF5* in HaCaT cells, we observed a decrease in both GATA-3 and FLI-1 at transcript and protein levels (Supplemental Figure S5; Supplemental Figure S8d). We confirmed by reporter assay that elevating ELF5 expression significantly decreases promoter activities for both GATA-3 and FLI-1 and both are targets of ELF5 in keratinocytes (*p value < 0.05, Fig. 5f).

### Modulation of FLI-1 and GATA-3 expression in ELF5-KO cells rescues keratinocytes ability to differentiate, inhibits cell migration and tumourigenicity

**Fig. 6:**
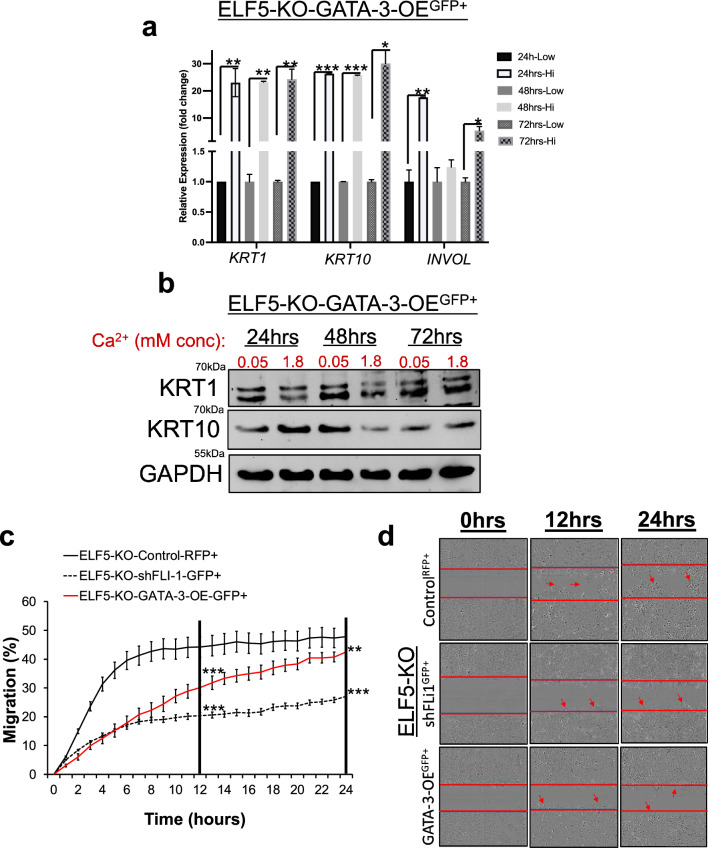
Overexpression of GATA-3 and knockdown of FLI-1 in ELF5-KO cells inhibits cell motility and rescues keratinocyte differentiation. Using CRISPR/Cas9 HaCaT-ELF5 knockout cells (ELF5-KO) cell variants. (**a–b**) RT-qPCR and western blot analysis of ELF5-KO-GATA-3-OE^GFP+^ cells during calcium induced keratinocyte differentiation, in vitro (low: 0.05 mM; high: 1.8 mM); increase in GATA-3 expression rescues the ability of ELF5-KO cells to re-differentiate as seen by elevated expression of differentiation associated markers (*CYTOKERATIN 1* (*KRT1*), *KRT10,* and *INVOLUCRIN* (*INVOL*) (panel** a**) and KRT1 and KRT10 protein levels (panel **b**). Data are presented as mean ± SEM values from three experimental repeats. Data shown are from a single representative experiment from three independent experiments. Densiometric analysis shown in Supplemental Figure S8f. (**c-d**) Overexpression GATA-3 (ELF5-KO-GATA-3-OE^GFP+^; GATA-3-OE^GFP+^) and knockdown of shFLI-1(ELF5-KO-shFLI-1^GFP+^; shFLI-1^GFP+^) inhibit the ability of cells to migrate compared to ELF5-KO-Control^RFP+^ cells. Representative images and data are presented as mean ± SEM values from five experimental repeats per group. Red line: original wound edge; red arrows: migrating cells. *p < 0.05, **p < 0.01, ***p < 0.001; unpaired Student’s *t*-test.

To determine whether the effects observed in ELF5‑KO keratinocytes (Figs. 2, 4) are potentially driven by deregulated expression of FLI‑1 and GATA‑3 (Fig. 5); using fluorescence-activated cell sorting (FACS), we subsequently performed rescue and knockdown experiments in which GATA‑3 was re‑expressed and FLI‑1 was silenced in ELF5‑KO cells using lentivirals (Supplemental Figure S6). Following FACS isolation of ELF5‑KO variants; we confirmed the successful overexpression of GATA-3 (ELF5-KO-GATA-3-OE^GFP+^) and knockdown of FLI-1 (ELF5-KO-shFLI-1^GFP+^) at transcript and protein levels (Supplemental Figure S7; Supplemental Figure S8e). Next, we assessed the ability of ELF5-KO-GATA-3-OE^GFP+^to differentiate after the re-expression of GATA-3 using calcium induced keratinocyte differentiation, in vitro assay. We observed that ELF5-KO-GATA-3-OE^GFP+^ cells were able to re-differentiate as seen by elevated transcript expression of differentiation markers (*CYTOKERATIN 1 (KRT1), KRT10 and INVOLUCRIN (INVOL*); *p value < 0.05; **p value < 0.01; ***p value < 0.001) and re-expression of KRT1 and KRT10 proteins, overtime (Fig. 6a,b; Supplemental Figure S8f.). We then assessed the migratory abilities of ELF5-KO-GATA-3-OE^GFP+^ and ELF5-KO-shFLI-1^GFP+^ cells. We observed that both loss of FLI-1 and overexpression of GATA-3 in ELF5-KO cells, significantly inhibited the migratory abilities of these cells compared to ELF5-KO-Control^RFP+^ cells overtime (**p value < 0.01; ***p value < 0.001, Fig. 6c,d).

**Fig. 7:**
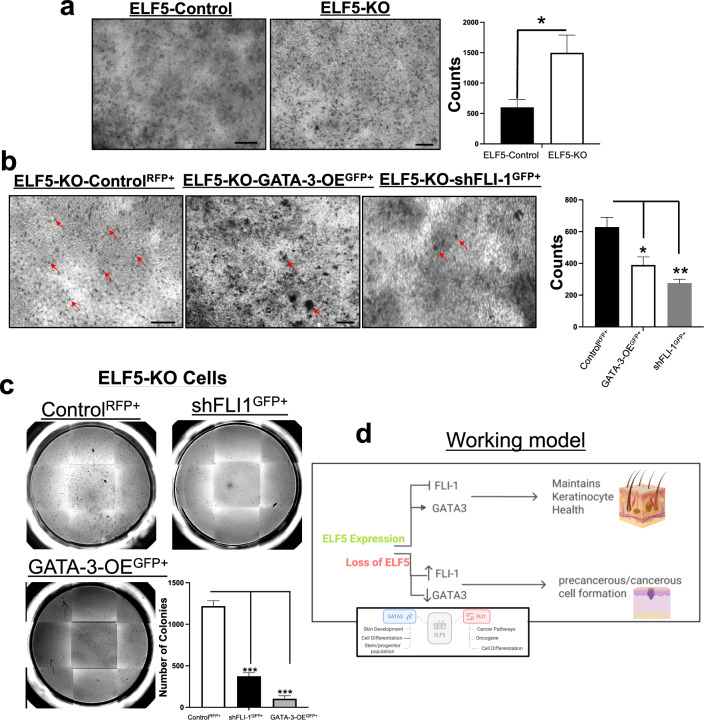
Modulation of GATA-3 and FLI-1 expression in ELF5-KO cells potentially inhibit tumourigenicity of keratinocytes. (**a–b**) Transwell assay; loss of ELF5 (CRISPR/Cas9 HaCaT-ELF5 knockout cells; ELF5-KO) significantly increases the ability of cells to migrate compared to CRISPR/Cas9 HaCaT-ELF5-Control cells (panel a). ELF5-KO variant cells: both increased expression of GATA-3 (ELF5-KO-GATA-3-OE^GFP+^; GATA-3-OE^GFP+^) and inhibition of FLI-1 (ELF5-KO-shFLI-1^GFP+^; shFLI-1^GFP+^) leads to significant inhibition of cell migration capabilities compared to ELF5-KO-Control^RFP+^ cells (red arrows, panel b). Data are presented as mean ± SEM values from three experimental repeats per group. (**c**) Soft agar assay: both knockdown of FLI-1 (ELF5-KO-shFLI-1^GFP+^; shFLI-1^GFP+^) and overexpression of GATA-3 (ELF5-KO-GATA-3-OE^GFP+^; GATA-3-OE^GFP+^) in ELF5-KO cells results in the inhibition of tumourigenicity of cells compared to ELF5-KO-Control^RFP+^ (Control^RFP+^) cells. Data are presented as mean ± SEM values from three experimental repeats per group. (**d**) The involvement of ELF5 as an important regulator of both GATA-3 and FLI-1, which is required to maintain keratinocyte homeostasis (cell differentiation, proliferation) and if deregulated can lead to the initiation of cancerous cell transformation. *p < 0.05, **p < 0.01, ***p < 0.001; unpaired Student’s *t*-test. Scale bars: 100 μm. Image created using Biorender.com.

Thereafter, we assessed the migratory capabilities of ELF5-KO cells using Transwell assay. We observed that a significant number of cells were able to migrate in ELF5-KO cells compared to ELF5-Control cells (*p value < 0.05; Fig. 7a). In addition, we assessed the migratory capabilities of ELF5-KO cell variants after overexpression of GATA-3 and knockdown FLI-1. We observed that in both ELF5-KO-GATA-3-OE^GFP+^ and ELF5-KO-shFLI-1^GFP+^ cells significantly lost the ability to migrate compared to ELF5-KO-Control^RFP+^ cells (*p value < 0.05; **p value < 0.01, Fig. 7b). Furthermore, to assess the tumourigenicity and invasion capabilities of ELF5-KO cell variants, we performed soft agar assay, which closely mimics the 3D cellular environment of cancer cells, in vivo^34^. We observed that there were significantly reduced number of colonies formed in ELF5-KO-GATA-3-OE^GFP+^ and ELF5-KO-shFLI-1^GFP+^ cells compared to ELF5-KO-Control^RFP+^ cells (***p value < 0.001, Fig. 7c).

## Discussion

Our findings on the role of ELF5 as a potential tumour suppressor in the skin are the first to our knowledge to be reported. We have identified a previously undescribed role for ELF5 in the control of skin cancer formation and we have shown that (i) ELF5 expression is significantly reduced in cSCC tissues and in cancerous cell lines (A431 and SCC-9) at both transcript and protein levels compared to healthy skin tissue and cells; (ii) CRISPR-Cas9 mediated knockout of ELF5 in immortalised human keratinocytes significantly enhances the ability of cells to proliferate, migrate and are more tumourigenic, in vitro. Furthermore, loss of ELF5 in keratinocytes, inhibits the ability of these cells to differentiate properly, in vitro. (iii) ELF5 regulates the balance of proliferation and differentiation in keratinocytes and prevents transformation of keratinocytes in part by regulating GATA-3 and FLI-1.

Analysis of the endogenous expression of ELF5 in healthy human tissue shows abundant nuclear expression in both basal and suprabasal layers. In cSCC tissue and cancerous cell line ELF5 is dramatically reduced (Fig. 1a,d). This data suggests that ELF5 is required potentially for regulating gene targets associated with stem/progenitor cells, proliferation, and differentiation during skin development and homeostasis and lost or reduced expression of ELF5 can cause the deregulation of these processes leading to the initiation and development of precancerous cells to cSCC. These findings are consistent with ELF5 known role in other epithelial tissues where ELF5 has been shown to inhibit migration and invasion of breast cancer cells by regulating CD24 expression^35^. A dramatic decrease in ELF5 levels are observed in the kidney, which is one of the highest ELF5-expressing tissues in healthy state, in cancer ^7^ as well as being characterized as a tumour suppressor in the kidney and bladder^36,37^. Recently, ELF5 has been proposed as a breast-specific biological clock to identify women at higher risk of breast cancer^38^. Furthermore, loss of ELF5 expression allows Snail2 to induce EMT and increases Snail2-dependent stem and progenitor activities in the mammary gland ^13^ and is associated with increased triple-negative breast cancer tumour initiation, progression and metastasis^14^. Our functional analysis supports these previous studies that loss of ELF5 can lead to inhibited differentiation and enhanced proliferation, migration and potentially tumourigenicity (Figs. 2, 4, 6, 7). Furthermore, overexpression of ELF5 can inhibit these effects in HaCaT and cancerous cell lines A431 and SCC-9 (Figs. 2, 3). Our findings are also supported by other studies showing that overexpression of ELF5 inhibits migration and invasion of ovarian cancer cells^39^. This data suggests that ELF5 acts as a molecular switch which is important for normal keratinocyte differentiation processes and downregulation of ELF5 expression could be an early diagnostic marker to detect precancerous cell formation before malignant transformation and visible lesions. Moreover, targeting ELF5 in precancerous cells using small-molecule activators and/or gene therapy could enable the restoration of ELF5 function in high-risk patients. However, additional studies are required to further extrapolate normal to precancerous states/cutaneous skin cancer.

Interestingly, consistent with our previous findings in primary mice epidermal keratinocytes^11^ that both *CDK16* and *CCNE1,* are significantly up-and downregulated after modulating ELF5 expression in HaCaT and cancerous cells lines (Figs. 2, 3). Both *CDK16* and *CCNE1* are critical regulators of cell cycle progression, and their deregulation can lead to disease or cancer formation^40^. Furthermore, *CCND1* is significantly elevated in ELF5-KO cells and is downregulated when ELF5 is overexpressed in HaCaT and cancerous cell lines (Figs. 2, 3). Previous studies have shown that deregulation of Cyclin D1 expression results in loss of restriction point control leading to uncontrolled cell growth and progression of human cancers^41^. Also, downregulation of *CCND1* is important for keratinocytes to exit the cell cycle and initiate differentiation^42^. Our data suggest that ELF5 can act as a switch between proliferation and differentiation of keratinocytes, by balancing and regulating the transition of keratinocytes from proliferation to early differentiation, which if deregulated could potentially lead to initial transformation of keratinocytes.

We have also identified GATA-3 and FLI-1 as targets of ELF5 in keratinocytes. Consistent with previous findings^30,43^, we observed GATA-3 expression is significantly reduced in cSCC tissues analysed and after loss of ELF5 in keratinocytes (Fig. 5 and Supplemental Figure S4). Mouse models using skin-specific *Gata-3* knockout studies have shown that loss of *Gata-3* leads to death of mice shortly after birth due to a dysplastic and non-functional skin^44^. In adult mice, loss of *Gata-3* lead to loss of integrity of the epidermal compartment^28,44,45^. In human skin, reduced GATA-3 expression was found in psoriatic lesions, atopic dermatitis and psoriasis (increased proliferation, impaired differentiation, and defective barrier formation) ^46,47^, and silencing of *GATA-3* during differentiation of human primary keratinocytes decreased the expression of several early and late differentiation markers (including cytokeratin 1 and 10), signifying that GATA-3 is key regulator of keratinocyte differentiation^30^. In cancer, GATA-3 has been shown to be significantly reduced in expression in cSCC tissues compared to healthy tissues^26^. These studies are consistent with our findings when CRISPR-mediated loss of ELF5 in HaCaT cells leads to the inability of cells to differentiation properly as observed with a significant reduction of keratinocyte differentiation associated markers as well as reduced GATA-3 expression in our cells and tissue (Figs. 4, 5d,e; Supplemental Figure S4). These findings were further supported by our observations after re-expression of GATA-3 in ELF5-KO cells rescues the keratinocytes’ ability to differentiation again (Fig. 6a,b). However, elevated GATA3 expression has also been linked with poor survival in triple-positive breast cancer, urothelial carcinoma and metastatic bladder tumours^48–50^. This suggests that ELF5 regulation of keratinocytes differentiation is in part due to regulation and maintenance of GATA-3 expression and deregulation of this mechanism could be a prerequisite for precancerous cell and dysplastic skin formation.

Conversely, we found that FLI-1 was significantly upregulated after loss of ELF5 and cSCC tissue biopsies (Fig. 5; Supplemental Figure S4). FLI-1 was initially identified as a proto-oncogene activated by proviral integration in F-MuLV-induced erythroleukemia’s and later in Cas-Br-E-induced Non-T/B-cell and 10A1 stem cell like-induced leukaemia’s^51,52^. FLI-1 has been shown to act according to the cellular context, such as a transcriptional activator or suppressor to regulate genes involved in cell proliferation, survival or differentiation^23,24^. Fli-1 downregulation of Gata-1 in retinoblastoma inhibits erythroid differentiation^53^. While upregulation of Bcl-2 and Mdm2 by Fli1 blocks apoptosis^53,54^ and destabilizes p53 (via Mdm2), which plays a critical role in the initial transformation of erythroblasts by Friend virus^25^. Furthermore, FLI-1 has been found to be highly expressed in the majority of benign and malignant vascular tumours^55^.

Of interest, overexpression of FLI-1 in erythroblasts resulted in inhibition of differentiation^53^ where at lower expression, FLI-1 activated Epo-EpoR in erythroid cells resulting in differentiation^55^. Furthermore, shRNA-mediated knockdown of FLI-1 in murine and human erythroleukemic cells suppresses cell proliferation and induces differentiation via its target genes *Gata-1* and *Bcl2*^22^. This suggests that ELF5 suppression of FLI-1 in keratinocytes is important to maintain the ability of keratinocytes to differentiation and to prevent initial transformation of keratinocytes.

Our data show that loss of ELF5 inhibited the ability of keratinocytes to differentiation properly (Fig. 4) in part by reduced GATA-3 expression and potentially due to elevated FLI-1 expression in keratinocytes (Figs. 5, 6, 7d). This suggests that ELF5 regulation of GATA-3 and FLI-1 is required to prevent the disruption to the differentiation program in keratinocytes leading to the initial transformation of keratinocytes. Loss of ELF5 can cause keratinocytes to fail to complete the differentiation process, leading to precancerous cell formation and dysplastic epithelium in skin. Collectively, our data has identified ELF5 as an important regulator of both GATA-3 and FLI-1, which are required to maintain keratinocyte homeostasis and if deregulated can lead to cancer initiation and formation (Fig. 7d). However, additional analysis is required to identify the precise ELF5-mediated regulation of GATA-3 and FLI-1 pathways in keratinocytes.

Based on our data the development of a suitable delivery system to introduce ELF5 into cancer cells and overexpress ELF5 may therefore have therapeutic potential. Elevated FLI-1 has been shown to have a critical role in cell survival and proliferation of angiogenic cells^55^. Thus by targeting ELF5, may not only inhibit expression of FLI-1 but also inhibit proliferation in tumours, as well as cut off their blood supply. Our data underscores the potential high value of ELF5 as a promising therapeutic target for preventing cSCC formation and/or restoring homeostatic mechanisms in the skin. However, further analysis is needed to determine the extent to which the loss of ELF5 contributes to keratinocyte transformation. Better understanding of the underlying mechanisms by which ELF5 regulates gene expression and consequently cell activity will provide new knowledge that can be translated into long-term benefits for patients with precancerous lesions to cSCC.

In summary, our data reveal that ELF5 is an important regulator of preventing initial transformation of keratinocytes in part by regulating GATA-3 and FLI-1. GATA-3 is required for differentiation and SC maintenance^29,30^. While FLI-1 expression levels dictate if differentiation takes place (FLI-1^Low^) or tumourigenesis (FLI-1^High^)^55^. Our data highlights the intricate interplay between ELF5, GATA-3 and FLI-1 in keratinocytes, which further adds to the complexity to keratinocyte cell fate determination (Fig. 7d). Our findings provide interesting evidence that ELF5 represents a potentially powerful candidate therapeutic target, which warrants further exploration. Nevertheless, additional studies such as in vivo tumour-formation assays are essential to substantiate our in vitro findings. Such studies will be an important foundation for further evaluation of the role of ELF5 as a regulator of pathway activities and could lead to identifying its importance in broader areas of research, including stem cells, regenerative medicine, and ageing.

## Material and methods

### Human tissue

Human healthy skin and cSCC tissue samples were obtained from donors (*n* = 3 each; 47–95 years old) undergoing face-lift or biopsy removal (face). Tissue was obtained with full written informed consent from all subjects and/or their legal guardian(s) adhering to the Declaration of Helsinki principles, following ethical and Nottingham Trent University institutional approval under Human Tissue Act guidelines at host institute.

### Immunohistochemistry

For immunohistochemical analysis, 4% paraformaldehyde-fixed cryostat sections (10 μm) were blocked with 0.2% Triton-X-100/PBS, 5% fetal calf serum, 2% bovine serum albumin and 10% normal goat and/or donkey serum (Merck, UK) and incubated with primary antibodies against ELF5, KRT15 and FLI-1 (Supplemental Table S1) overnight at 4 °C. The following day, slides were incubated with the corresponding Alexa-Fluor-488 or Alexa-Fluor-A555 (Supplemental Table S1) secondary antibodies for 1 h at room temperature. Incubation steps were interspersed with wash steps with 0.2% Triton-X-100/PBS.

Formalin fixed (10%) cSCC paraffin tissue sections (10 μm) were deparaffinized, rehydrated and subjected to antigen retrieval by boiling for 1 h in Tris–EDTA pH 9.0 as described previously^10^, followed by incubated with primary antibodies against ELF5 and FLI-1 (Supplemental Table S1) overnight at 4 °C. The following day, slides were incubated with the corresponding Alexa-Fluor-488 secondary antibodies for 1 h at room temperature. Incubation steps were interspersed with wash steps with 0.2% Triton-X-100/PBS. Sections were mounted with mounting medium with DAPI. Images were taken using Leica THUNDER imager 3D cell culture (Leica, Germany).

### Cell culture and transfection

Human primary epidermal keratinocytes (HPEKs, freshly isolated) cells were generated from anonymized healthy female donors (47–51 years old) undergoing face-lift as described above and previously^56,57^. Post extraction, cells were grown in keratinocyte Basal Medium 2 (PromoCell), supplemented with Bovine Pituitary extract (BPE) 4 μl/ml, human epidermal growth factor (hEGF, 0.125 ng/ml), Insulin (recombinant human, 5 μg/ml), hydrocortisone (0.33 μg/ml), Epinephrin (0.39 μg/ml), transferrin (recombinant human, 10 μg/ml) and calcium chloride (0.06 mM) at 37 °C, 5% CO_2,_ until 60–70% confluent and collected for RNA extraction.

Immortalised human keratinocytes (HaCaT; 300493, 2B Scientific)^58^ and A431 (CRL-1555, ATCC)^59^ were grown in DMEM (Fisher, UK) respectively, supplemented with 10% heat-inactivated fetal bovine serum (Fisher, UK), 100U/ml penicillin (Fisher, UK), 100 µg/ml streptomycin (Fisher, UK), and 2 mM L-glutamine (Fisher, UK), at 37 °C, 5% CO_2,_ respectively, until 60–70% confluent. SCC-9 cell line (89062003, Merck)^60^ was cultured in DMEM/F12 (Fisher, UK) 10% heat-inactivated fetal bovine serum, 100U/ml penicillin, 100 µg/ml streptomycin, 2 mM L-glutamine, 0.4 μg/ml hydrocortisone (Fisher, UK) and 0.5 mM Sodium Pyruvate (Fisher, UK) at 37 °C, 5% CO_2_ until 60–70% confluent.

Cells were transfected with pControl plasmid (pEGFP-N1, CloneTech, UK) or pELF5 overexpression plasmid (pELF5-OE, RG231905—Isoform 3, Insight Biotechnology, UK) using Lipofectamine 3000 (Fisher, UK) following manufactures instructions as done previously^61^. In vitro inducible expression of ELF5 isoforms 1, 2 and 3 have been to have similar phenotypic and transcriptional effects^7^. Cells were harvested 48-h post-transfection for RNA and protein analysis. For cell migration, media was replaced after 48-h post-transfection and cells were then used for downstream analysis.

### TaqMan copy number variation assay

TaqMan copy number assays was performed using TaqMan MGB probe (ThermoFisher, UK) and analysed using CopyCaller software (ThermoFisher, UK) following manufacturer instructions to determine copy number of ELF5 in immortalised human keratinocytes (HaCaT) cell line.

### Production of lentiviruses

HEK293T cells were cultured in DMEM (ThermoFisher, UK) supplemented with heat-inactivated 10% fetal bovine serum, 1% L-glutamine and 5% non-essential amino acids (Fisher, UK) at 37 °C, 5% CO_2_ as described previously^11^. In brief, for production of viral particles for CRISPR-Cas9-Control, CRISPR-Cas9-mediated ELF5 knockout (KO), GATA-3 overexpression (GATA-3-OE), shFLI-1 (knockdown) and Control lentiviruses: at 40% confluence HEK293T cells were co-transfected with either Dharmacon™ All-in-one system SMARTvector Non-targeting hCMV-TurboGFP (CRISPR-Cas9 Control, Horizon, UK), Dharmacon™ All-in-one system SMARTvector Human Lentiviral shRNA ELF5 (CRISPR-Cas9 ELF5-KO, Horizon, UK; guide sequence: ‘5-AATTGATGCCAGTGTCTGT-3’; Precision Lenti-ORF® RFP Control, Lenti-ORF® GFP GATA-3 (RC219044L2, Insight Biotechnology, UK) and SMARTvector Human Lentiviral GFP shRNA FLI-1 (Horizon, UK) with packaging plasmid using TransLentiviral packaging kit (TLP5913, Horizon, UK) following manufacturer’s instructions. Cell culture medium containing viruses was collected at 24 h and 48 h and combined, followed by precipitation of the viral particles using 80 µg/ml polybrene (Fisher, UK) and 80 µg/ml chondroitin sulphate C (Merck, UK) for 18 h at 4 °C before centrifugation at 10,000xg. Titration of lentiviral particles was performed using RT-qPCR lentivirus titration kit (NBS Biological, UK) as per manufacturer’s instruction.

### CRISPR-Cas9-mediated knockout of ELF5 in keratinocytes

HaCaT cells were cultured in DMEM supplemented with 10% fetal calf serum (FCS) and 2% streptomycin/penicillin and grown at 37 °C with 5% CO_2_ as described above. For CRISPR/Cas-assisted gene editing, cells were transduced with either: CRISPR-Cas9 Control Lentivirus or CRISPR-Cas9 ELF5-Knockout Lentivirus (MOI of 20: 5.7 × 10^6^ viral titre/per ml). After 48 h of infection, the minimal lethal dose of puromycin (0.5 μg/ml, Fisher, UK) was added to select positive transduced cells. Following 9 days of puromycin selection, 5 × 10^4^ transduced cells were serially diluted from which single cell-derived clones were isolated and further expanded. To analyses the CRISPR/Cas mutation, the region of the gRNA-binding side was amplified by PCR. PCR products were cloned using Zero Blunt Cloning Kit (Fisher, UK) and sequenced to detect the mutation side (data not shown) and identified knockouts were confirmed by RT-qPCR and western blot analysis (Fig. 1f, g). Selected successful knockout Clone 1 (ELF5-KO) and associated control (ELF5-Control, Fig. 1f, g) were used for subsequent experiments.

### Transduction of ELF5-KO cell line with GATA-3 and FLI-1 viruses

Using ELF5-KO cell line: cells were infected a MOI of 20 for Control virus (7.8 × 10^5^ viral titre/per ml), a MOI of 20 for GATA-3 overexpression virus (4.4 × 10^9^ viral titre/per ml) and a MOI of 20 for shFLI-1 virus (2.06 × 10^7^ viral titre/per ml) for 48 h. These cells were then prepared for FACs Melody sorting as described below.

### Fluorescent activated cell sorting (FACS)/FACs melody

ELF5-KO cells were infected with GATA-3 overexpression^GFP+^, shFLI-1^GFP+^ (knockdown) and Control^RFP+^ lentiviruses for 48 h as described above. Cells were then cultured for 10 days post transduction. After 10 days, cells were detached and resuspended into a single cell suspension and filtered using a 40 μm filter. The cells were centrifuged down at 300xg for 10 min and resuspended in 1 ml of PBS. RFP + and GFP + cells were sorted using a BD FACSMelody™ Cell Sorter (Beckman Coulter, UK) and data was analysed using BD FACSChorus™ Software (Beckman Coulter, UK) (Supplemental Figure S6). FACs sorted RFP + and GFP + cells were cultured under normal HaCaT growth conditions (as described above) and used for subsequent experimental analysis. Images of RFP + and GFP + cells was captured using Incucyte® Live-Cell Imager (Sartorius).

### Growth curve assay

To assess if there were any effects on the growth potential of HaCaT cells after CRISPR-Cas9-mediated knockout of ELF5, a growth curve was performed. 5 × 10^3^ cells were seeded per six well. Cells were counted at 50 h and 100 h time points. Data from *n* = 3 experimental repeats per group and statistical analysis was performed using unpaired student’s *t-*test.

### Calcium induced keratinocyte differentiation, in vitro assay

For calcium induced keratinocyte differentiation analysis of CRISPR-Cas9 edited cells: ELF5-Control, ELF5-KO and ELF5-KO-GATA-3-OE^GFP+^ cell lines were utilized. Each cell line was differentiated using DMEM calcium free media (ThermoFisher, UK) supplemented with 10% chelated fetal bovine serum, 100U/ml penicillin, 100 µg/ml streptomycin, 2 mM L-glutamine and supplemented with either 0.05 mM (low) or 1.8 mM (high) calcium at 37 °C, 5% CO_2,_ for 24 h, 48 h and 72 h for further analysis.

### Reverse transcription quantitative real-time PCR (RT-qPCR)

Total RNA (cells and tissues) was isolated using the Zymo Direct-zol RNA kit (Cambridge Biosciences, UK). For paraffin embedded tissues, total RNA was extracted from whole tissue cSCC biopsies using Quick-RNA FFPE MiniPrep (Cambridge Biosciences, UK). For gene expression analysis, 100 ng of total RNA was converted into cDNA using the qPCRBIO cDNA Synthesis Kit system (PCR Biosystems, UK). Gene expression analysis was performed on QuantStudio 5 Real Time PCR System (ThermoFisher, UK). Gene expression was analysed using qPCRBIO SyGreen mix (PCR Biosystems, UK) at the following conditions: 95 °C for 2 min, followed by 40 cycles of denaturation (95 °C for 5 s), annealing and extension (30 s at temperature experimentally determined for each primer pair). RT-qPCR primers (Supplemental Table S2) were designed using Primer3 (https://primer3.ut.ee/) and further validated using UCSC genome browser (https://genome.ucsc.edu/) and NCBI primer blast (https://www.ncbi.nlm.nih.gov/tools/primer-blast/). Amplification differences between samples were calculated based on the Ct (^ΔΔ^Ct)^62^ method and normalized to human *ACTIN* gene (*ACTB*).

For calcium induced keratinocyte differentiation, relative gene expression was quantified using the ^ΔΔ^Ct method^62^. Ct values were normalized to the human *ACTB* gene housekeeping gene, and fold change values were calculated relative to the 24-h low calcium condition, which served as the reference sample/time-point for each target gene analysed.

Data from triplicates were pooled, standard error of the mean (± SEM) was calculated, and statistical analysis was performed using unpaired student’s *t-*test.

### Western blot

Whole cell protein lysates were extracted from cultured cells using RIPA lysis buffer (50 mm Tris–HCl, 1% NP-40, 0.25% sodium deoxycholate, 150 mm NaCl, and 1 mm EDTA; pH 7.4) and cOmplete ULTRA Protease Inhibitor Cocktail (Merck, UK). Western blot was performed as described previously^61^. In brief, 10 μg (micrograms) of protein was processed for western blot analysis, followed by membrane incubation with primary antibody overnight at 4 °C (Supplemental Table S1). Horseradish peroxidase tagged IgG antibodies were used as secondary antibodies (1:3000, ThermoFisher, UK, Supplemental Table S1). Antibody binding was visualized with an enhanced chemiluminescence system (SuperSignal West Pico Kit, ThermoFisher, UK) on the FL1000 iBright GelDoc Imager (ThermoFisher, UK). Blot imaging: all blots were imaged using the system’s Smart Exposure™ auto‑exposure technology, which automatically determines the optimal exposure time to prevent saturation and ensure consistent image quality across experiments. Images were captured with the instrument’s 9.1‑megapixel camera and exported in standard TIFF/PNG/JPEG formats without additional post‑processing. Densiometric analysis was performed using Image J (https://imagej.net/ij/) (Supplemental Figure S8). Data shown as ratio relative to GAPDH (arbuitry units, a.u.) with standard error of the mean (± SEM), and statistical analysis was performed.

### Flow cytometry

ELF5-Control, ELF5-KO and transiently transfected HaCaT cells with pELF5 overexpression (ELF5-OE) were cultured as described above. Cells were trypsinized, washed and fixed in 70% ethanol/PBS at − 20 °C for 30 min. Fixed cells were incubated with RNase A (100 μg/ml) for 30 min at 37 °C. Cells were subsequently incubated with 20 μg/ml Propidium Iodide (ThermoFisher, UK) for 30 min at 4 °C. The percentage of cells at distinct phases of the cell cycle was analysed by flow cytometry with a Beckman Coulter Gallios (Beckman Coulter, UK). For each sample, 10,000 events were collected and analysed on Beckman Coulter Kaluza Analysis Software (Beckman Coulter, UK) as done previously^11,12^.

### Scratch assay

CRISPR/Cas9 ELF5-Control, CRISPR/Cas9 ELF5-KO and transiently transfected HaCaT cells with pControl and pELF5 overexpression (ELF5-OE) were cultured as described above. Cells were grown to confluence on Incucyte® Imagelock 96-well Microplate (Sartorius) 24 h prior to scratch. Two hours before the scratched was induced the wells were supplemented with mitomycin C (final concentration of 10 µM, Fisher, UK). The scratch was set by using a Incucyte® Woundmaker Tool (Sartorius). Then the cells were transferred to the Incucyte® Live-Cell Imager (Sartorius). Images (Phase contrast) were taken with a 10 × air objective every 30 min for a total of 24 h. Analysis of the scratch closing was done using Incucyte® Base Analysis Software (Sartorius). Data from *n* = 5 per time point were pooled, and statistical analysis was performed using unpaired student’s *t-*test.

### RNA-sequencing analysis

ELF5-Control and ELF5-KO cells were cultured as described above, and total RNA was extracted using the Zymo Direct-zol RNA kit. Samples were sent to Novogene (Cambridge, United Kingdom) for RNA sequencing using Human mRNA Sequencing NovaSeq X Plus Series (PE150). Quality of RNA reads was assessed using Fastqc (v0.12.1) for presence of adapters and low-quality reads. Reads were aligned to the GRCh38.p14 genome using STAR (v2.7.10b)^63^ and read counts for each gene was evaluated using Feature Counts from the SubRead package (v2.0.1). Raw counts were input to Rstudio (v4.2.2) for further analyses. Normalization and differential gene expression analysis between groups were performed using EdgeR (v3.38.4). Genes with a Benjamini–Hochberg false discovery rate (FDR) < 0.05 were considered significant. Normalized counts were used to generate heatmaps for gene expression level using pHeatmap package (v1.0.12) and differentially expressed genes were plot as a Volcano plot using Enhanced Volcano package (v1.14.0). Gene ontology analysis was performed with significant enrichment threshold of P < 0.01 and Q < 0.05 using Cluster Profiler package (v4.4.4) and annotated using the org.Hs.e.g.db database (v3.15.0). Barcode plots were generated using the Limma package (v3.52.4) and normalized enrichment score calculated using the fgsea package (v1.24.0). Principal component analysis (PCA) was conducted using PCA tools package (v2.14.0) (Supplemental Fig. 3).

### Promoter reporter assay

To generate a GATA-3 and FLI-1 reporter constructs, promoter region (− 1 kb) were initially analysed for potential binding sites for ELF5 using Ciiider software (www.ciiider.org) as described previously^64^. PCR promoter fragments from human genomic DNA using Phusion High-Fidelity PCR Master Mix (Fisher, UK) were amplified using the following primers; pGATA-3-Luc: 5’-ttttggtaccGGTTGGGGTCGGTGCAGA-3’ and 5’-aaaactcgagACGCTCAGTGTAGACGGAC-3’ and pFLI-1-Luc: 5’-ttttggtaccCGCCTCAGGAAAAGCAAAGA-3’ and 5’-aaaactcgagAAGAGCTAGCCGGGAACAAT. These PCR fragments were purified and cloned into pGL3-basic vector (Promega, UK) at KpnI and XhoI sites (underlined sequences). For reporter assay^65^, HaCaT cells were seeded into 96-well plates (10^4^ cells per well) a day prior to transfection. Cells were co-transfected with ELF5 overexpressing plasmid (pELF5-OE) or control plasmid (pCDNA3 plasmid, 120 ng/well) along with one of the reporter constructs (pFLI-1-Luc or pGATA-3-Luc; 60 ng/well) using Lipofectamine 3000 (0.5 μl/well). Forty-eight hours after transfection, relative luciferase activities were measured using Dual-Glo luciferase assay system (Promega, UK) as per the manufacturer’s instruction. Luminescence was measured using CLARIOStar plate reader (BMG Labtech, Ortenberg, Germany). Data from three independent experiments were normalized to the pnull-Renilla (20 ng/well, Promega, UK) construct activity. Data was pooled, ± SEM was calculated, and statistical analysis was performed using unpaired Student’s *t-*test.

### Transwell assay

Transwell assay was performed as described previously^66^. In brief, CRISPR-Cas9 edited ELF5-control, ELF5-knockout (KO) as well as FACs sorted ELF5-KO cell variants: ELF5-KO-Control^RFP+^, ELF5-KO-GATA-3-OE^GFP+^ and ELF5-KO-shFLI-1^GFP+^ were grown (as described above) and detached, resuspended in serum free DMEM media and seeded (2 × 10^5^ cells/ml) to the upper chambers of Transwell equipment with 8-µm polycarbonate membrane filter (Corning, Inc.). The lower chambers were filled with DMEM containing 10% FBS. After incubation for 24 h at 37˚C, 5% CO_2,_ the migrated keratinocytes were stained with 0.5% crystal violet for 15 min at room temperature and counted using an optical inverted microscope (magnification, × 100). Data from n = 3 per group were pooled and statistical analysis was performed using unpaired student’s *t-*test.

### Soft agar assay

Growth and survival of HaCaT cell variants was determined as previously described^67–69^. Briefly, ELF5-KO-Control^RFP+^, ELF5-KO-GATA-3-OE^GFP+^ and ELF5-KO-shFLI-1^GFP+^ cells were grown as described above, followed by trypsinization and resuspension into single cells (10^4^ cells) in 2 mL medium containing 0.6% agar and 2mls of DMEM media containing 10% fetal calf serum. Cell suspensions were added to 1.2% agar layers in 96-well plates. Wells were examined immediately after plating showing only single cells. Cells were cultured for 28 days as described previously^68^. Soft agar colonies were stained at day 28 with NBT (nitroblue tetrazolium chloride solution, 1 mg/ml stock solution in 1 × PBS, Fisher, UK) and incubated overnight with stain at 37 °C, 5% CO_2_. Once colonies are stained, images were taken using Leica THUNDER imager 3D cell culture (Leica, Germany) and colonies counted using Image J (https://imagej.net/ij/) analysis software. Data from n = 5 per group were pooled and statistical analysis was performed using unpaired student’s *t-*test.

## Supplementary Information

Supplementary Information 1.

Supplementary Information 2.

Supplementary Information 3.

Supplementary Information 4.

## Data Availability

The data that support the findings of this study are openly available in Gene Expression Omnibus Datasets at https://www.ncbi.nlm.nih.gov/gds, reference number: GSE295801.
