# Supplementary material for: Interplay of ELF5, GATA-3 and FLI-1 regulate malignant transformation of keratinocytes
Source: Sci Rep. 2026 Jul 16;16:25881. doi: 10.1038/s41598-026-59939-0 (PMC13486807; doi:10.1038/s41598-026-59939-0)

Figure 1d: ELF5 31kDa

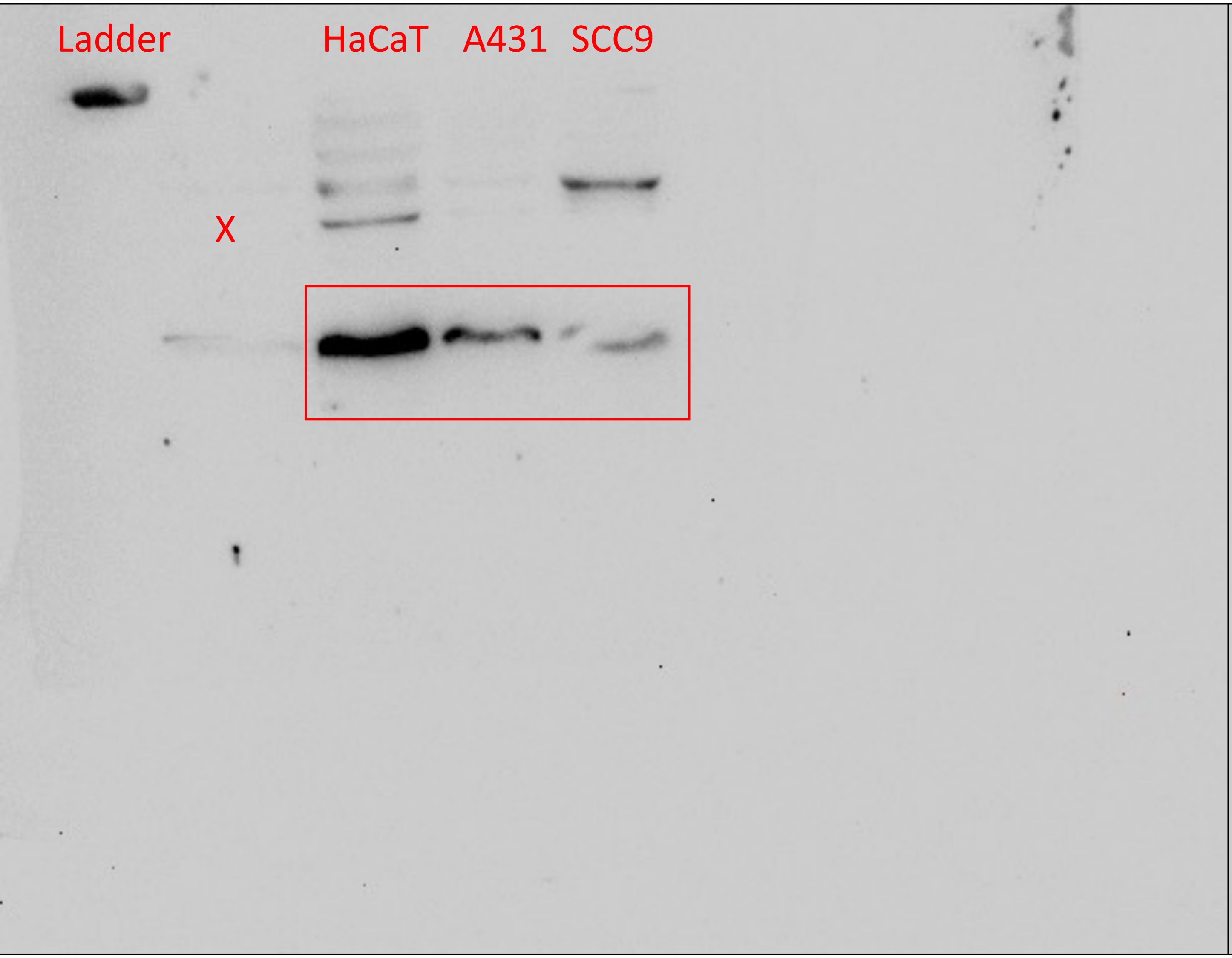

Figure 1d: GAPDH 36kDa

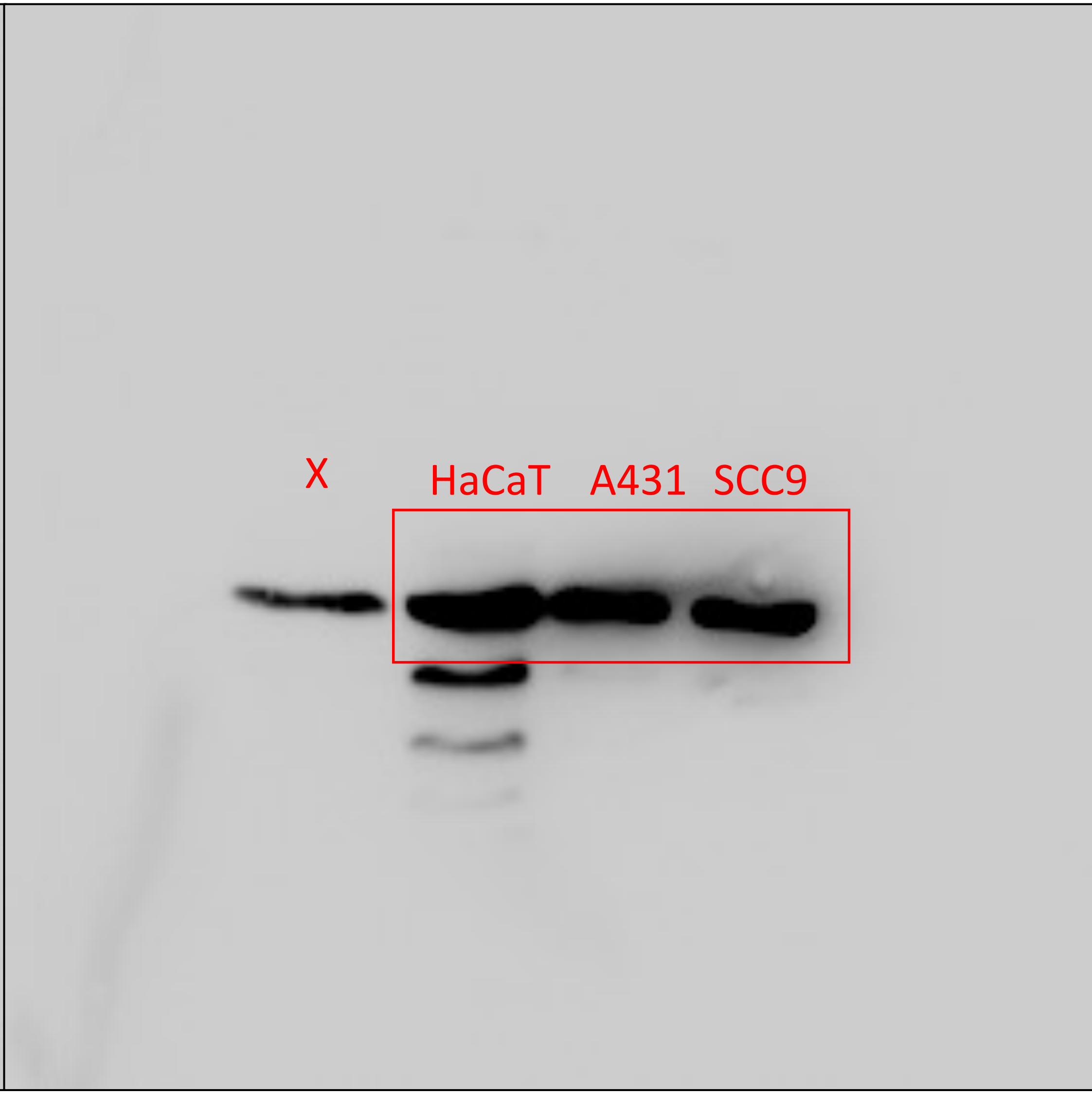

Figure 1g: ELF5 31kDa Clone 1 and 2

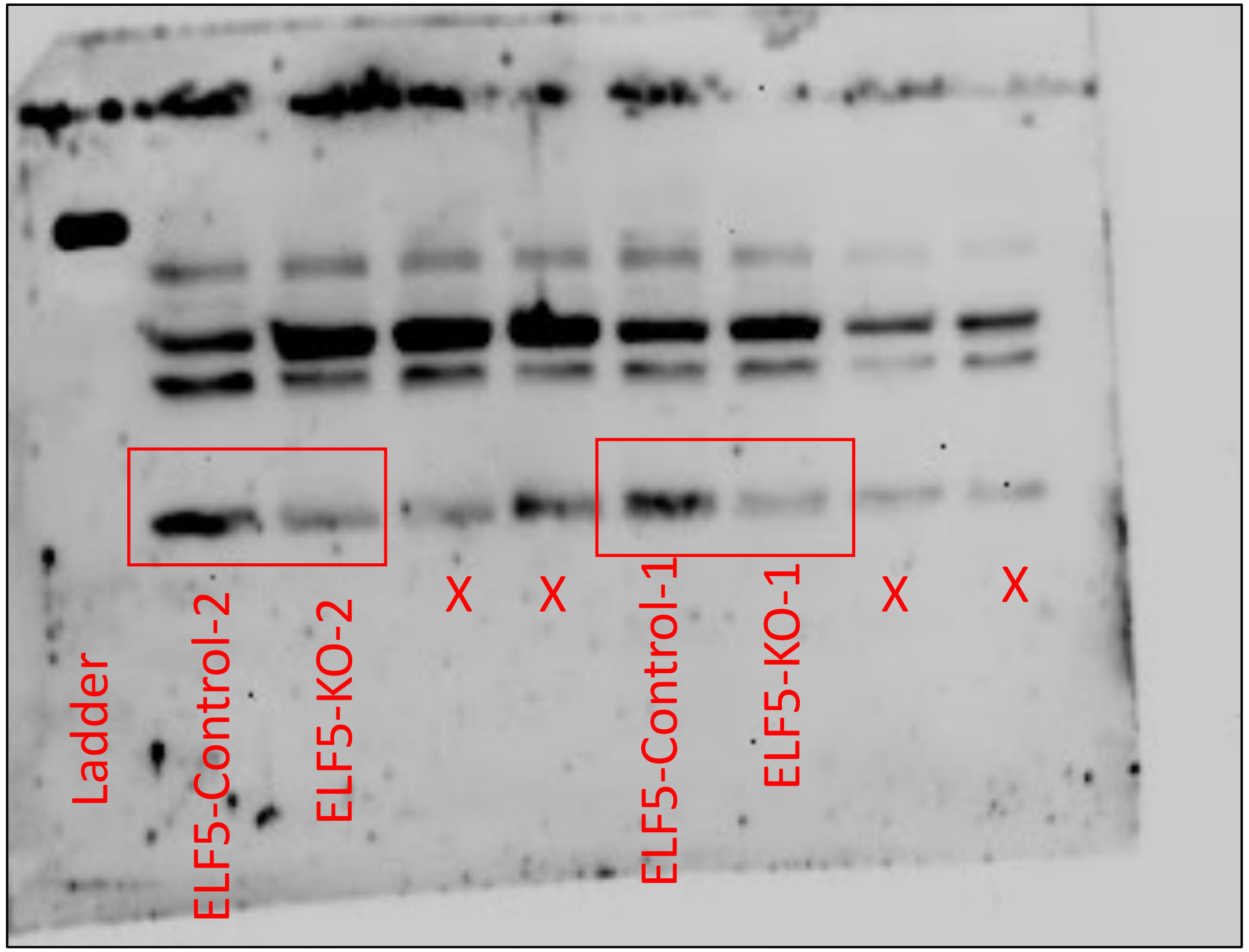

Figure 1g: GAPDH 36kDa Clone 1 and 2

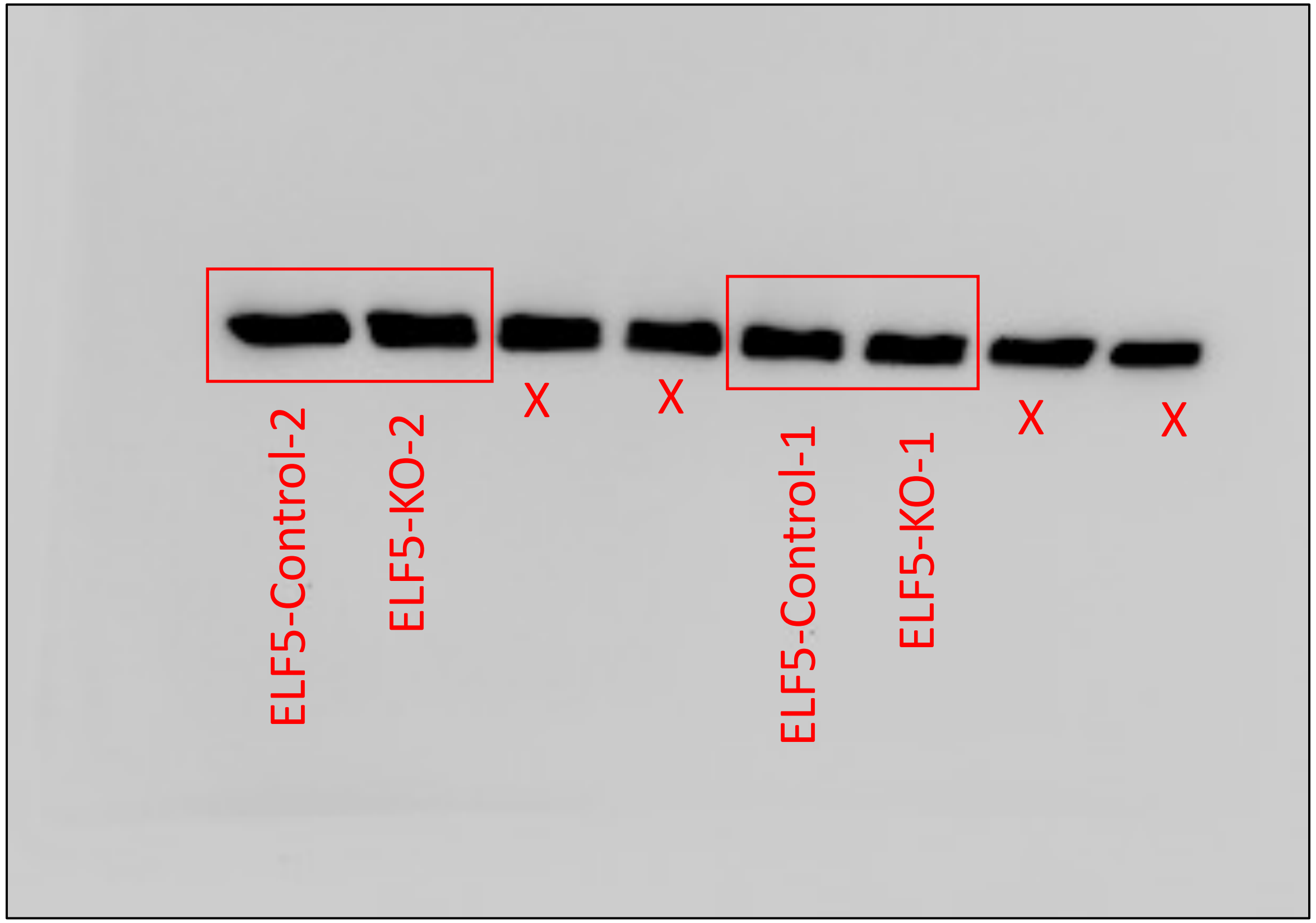

Figure 4c: CRISPR-ELF5-Control Low vs Hi calcium induced differentiation
ELF5 31 kDa (Panel A)
CYTOKERATIN 1 (KRT1) – 66kDa (Panel B)
GAPDH - 36kDa (Panel C)
KRT10 – 60kDa (Panel D)

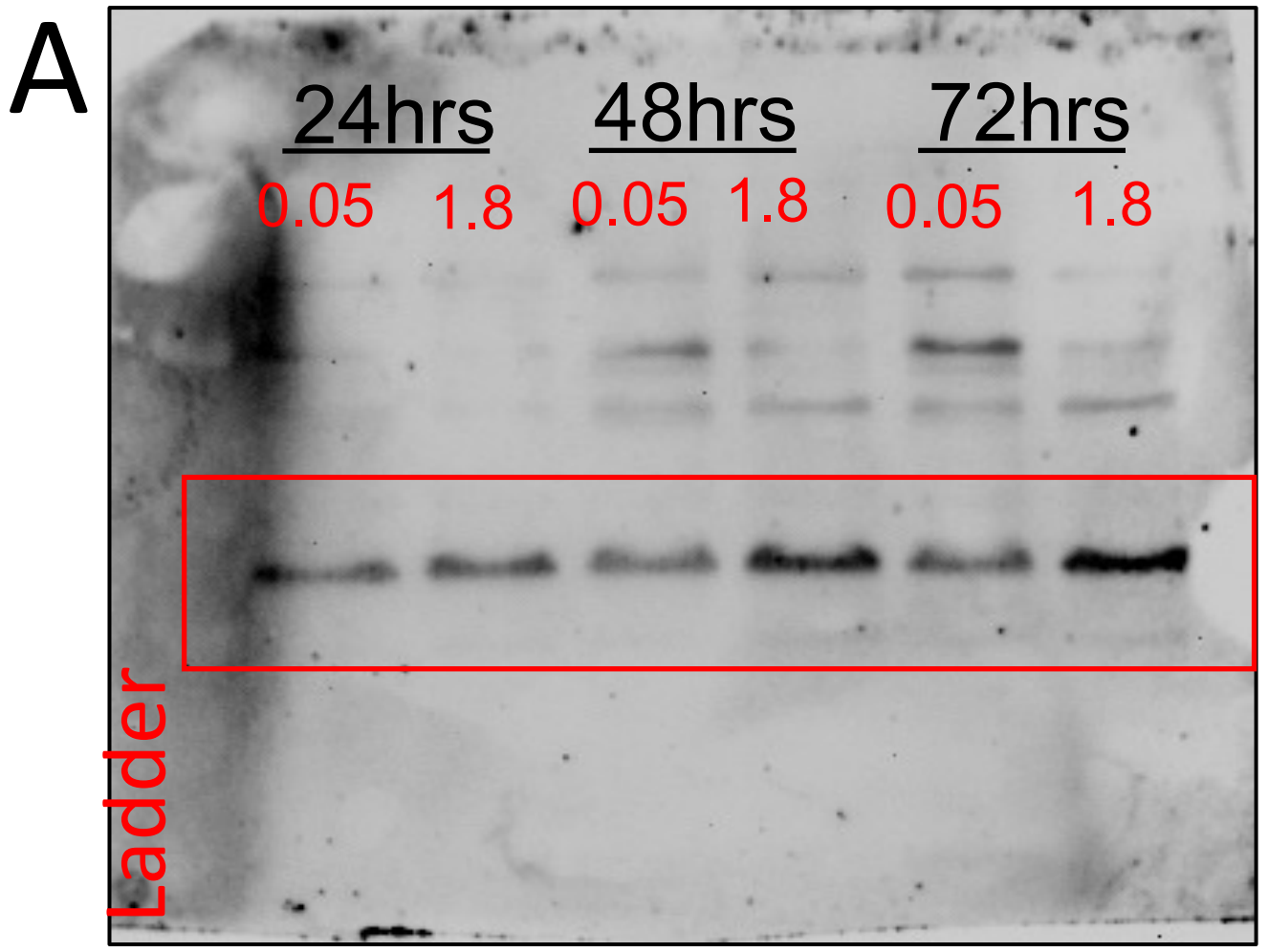

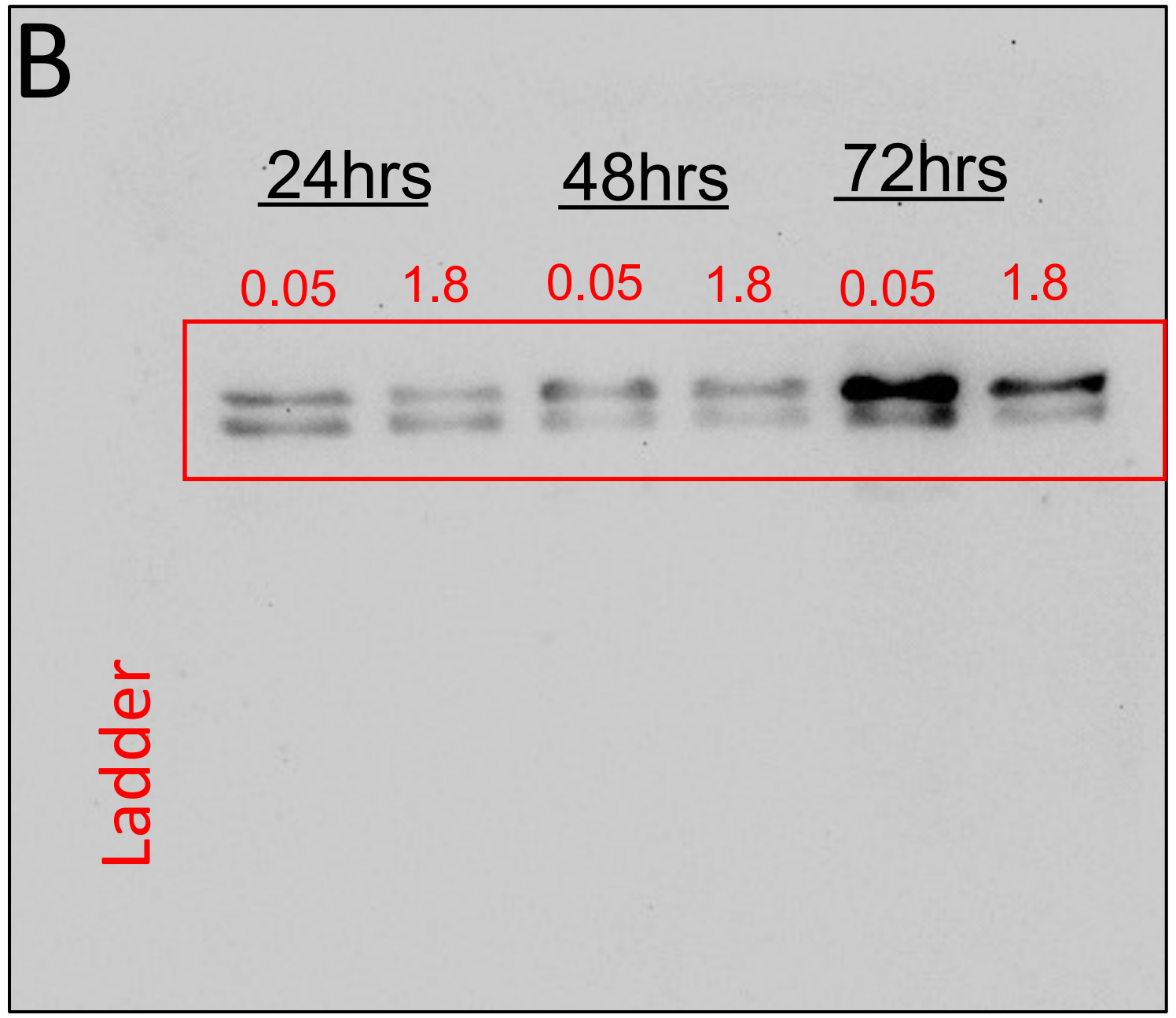

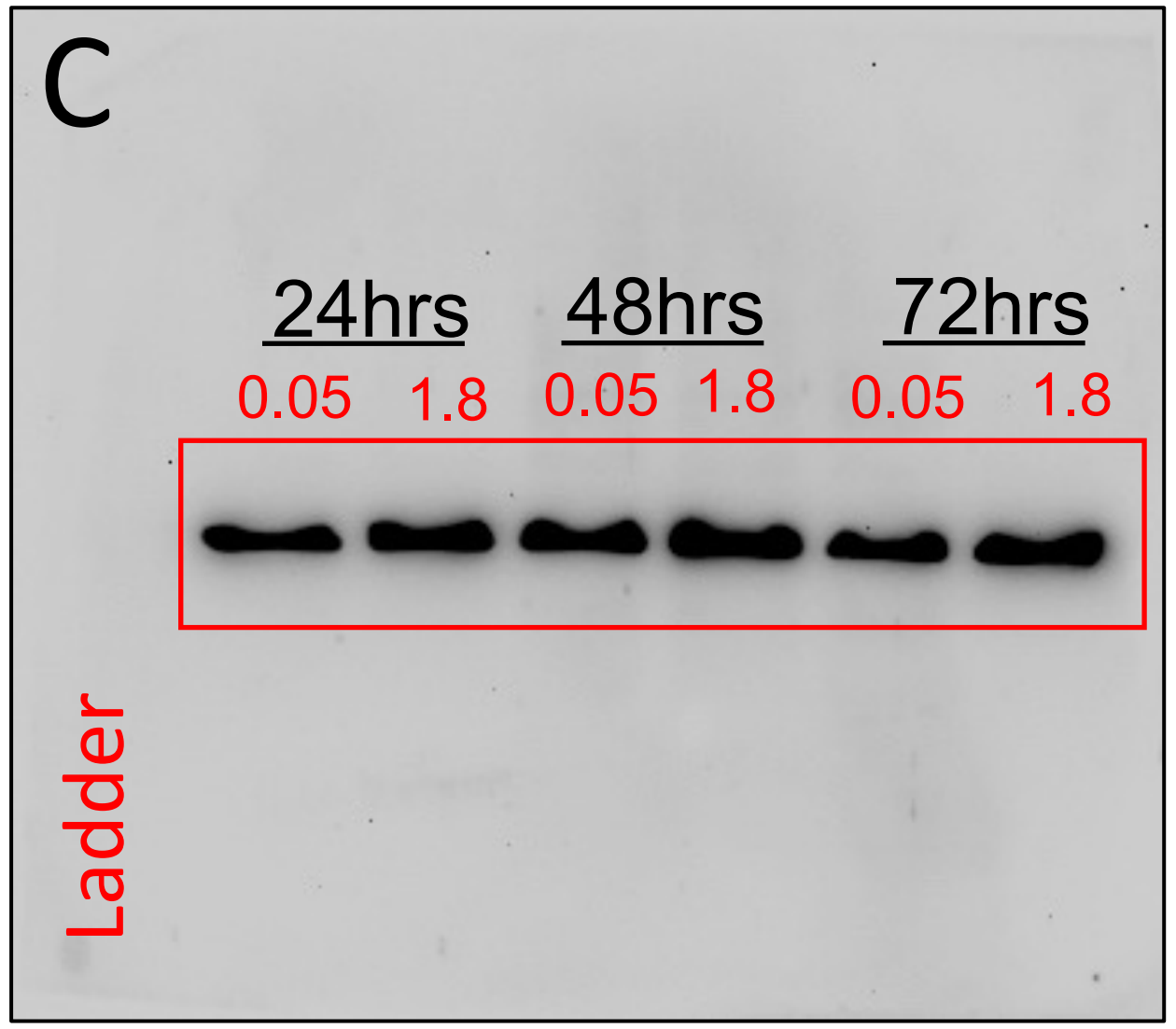

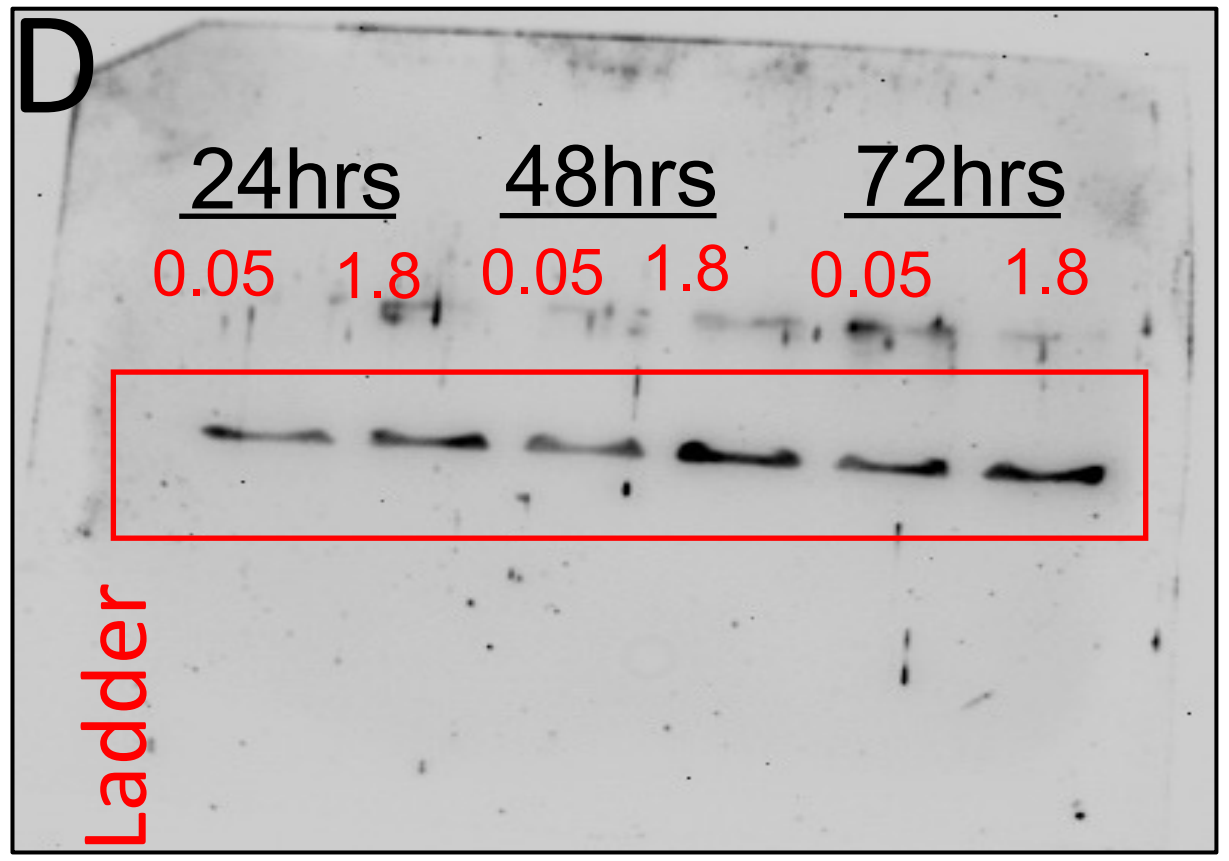

Figure 4d: CRISPR-ELF5-KO Low vs Hi calcium induced differentiation

ELF5 31 kDa (Panel A)

CYTOKERATIN 1 (KRT1) – 66kDa (Panel B)

GAPDH - 36kDa (Panel C)

KRT10 – 60kDa (Panel D)

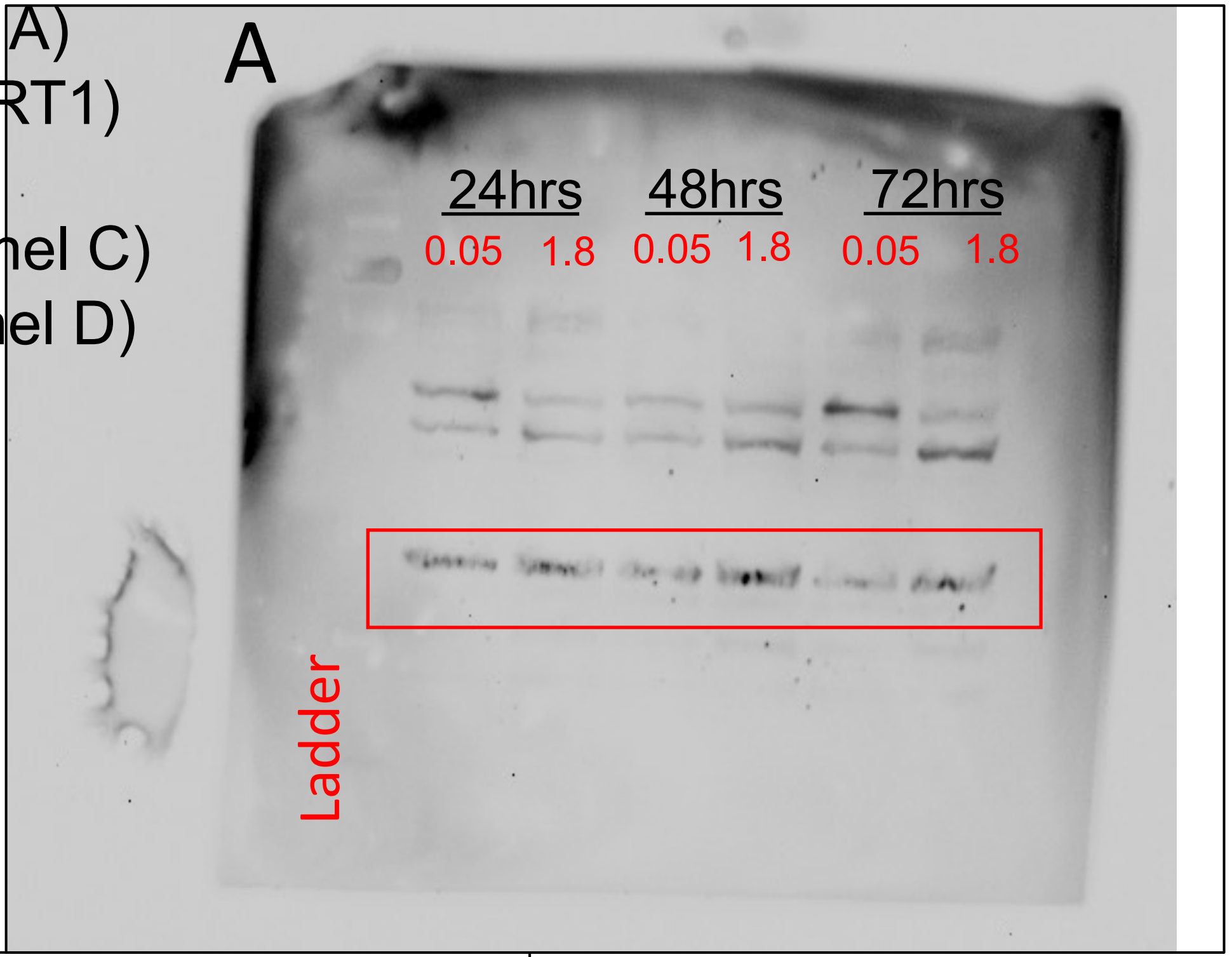

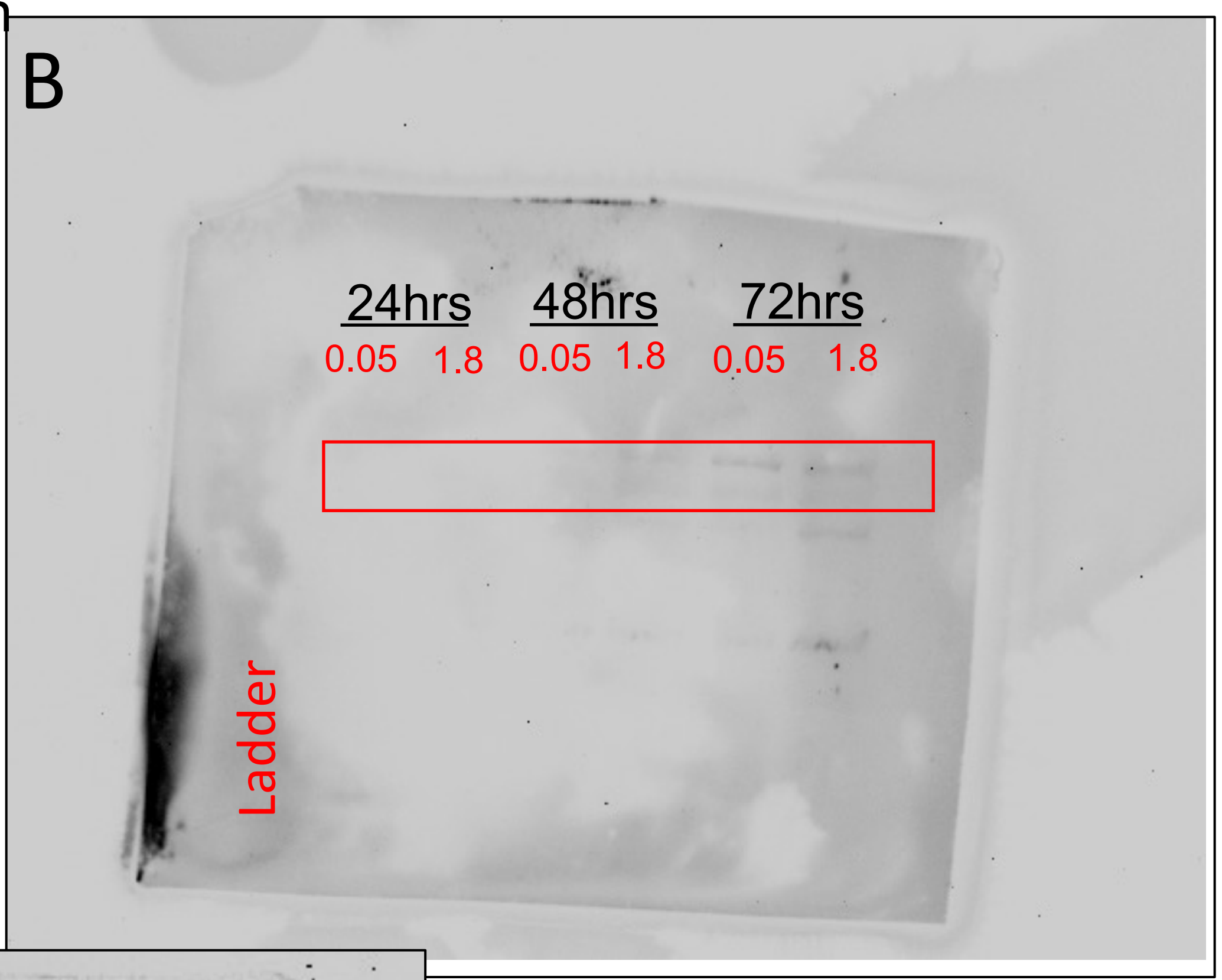

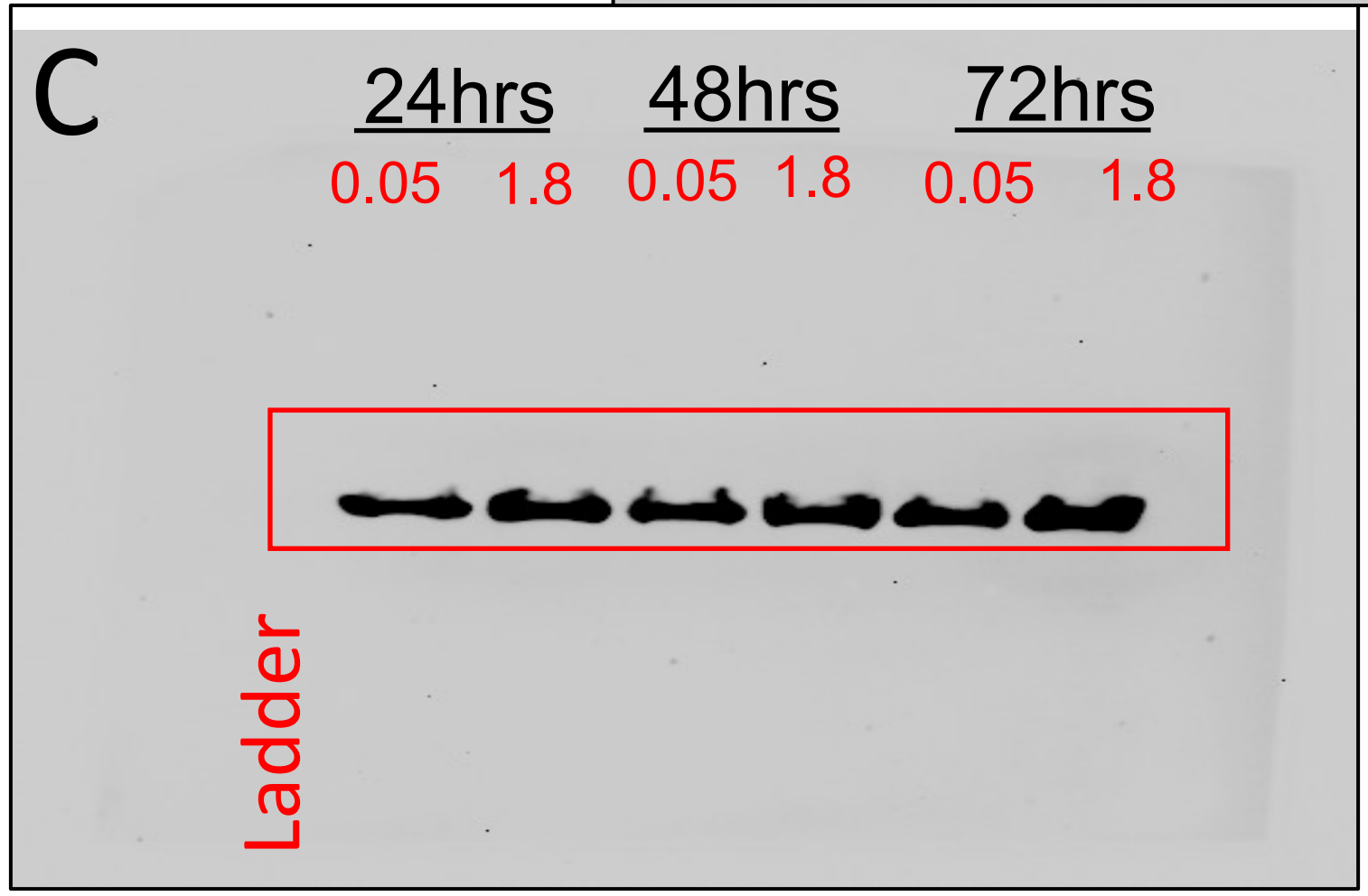

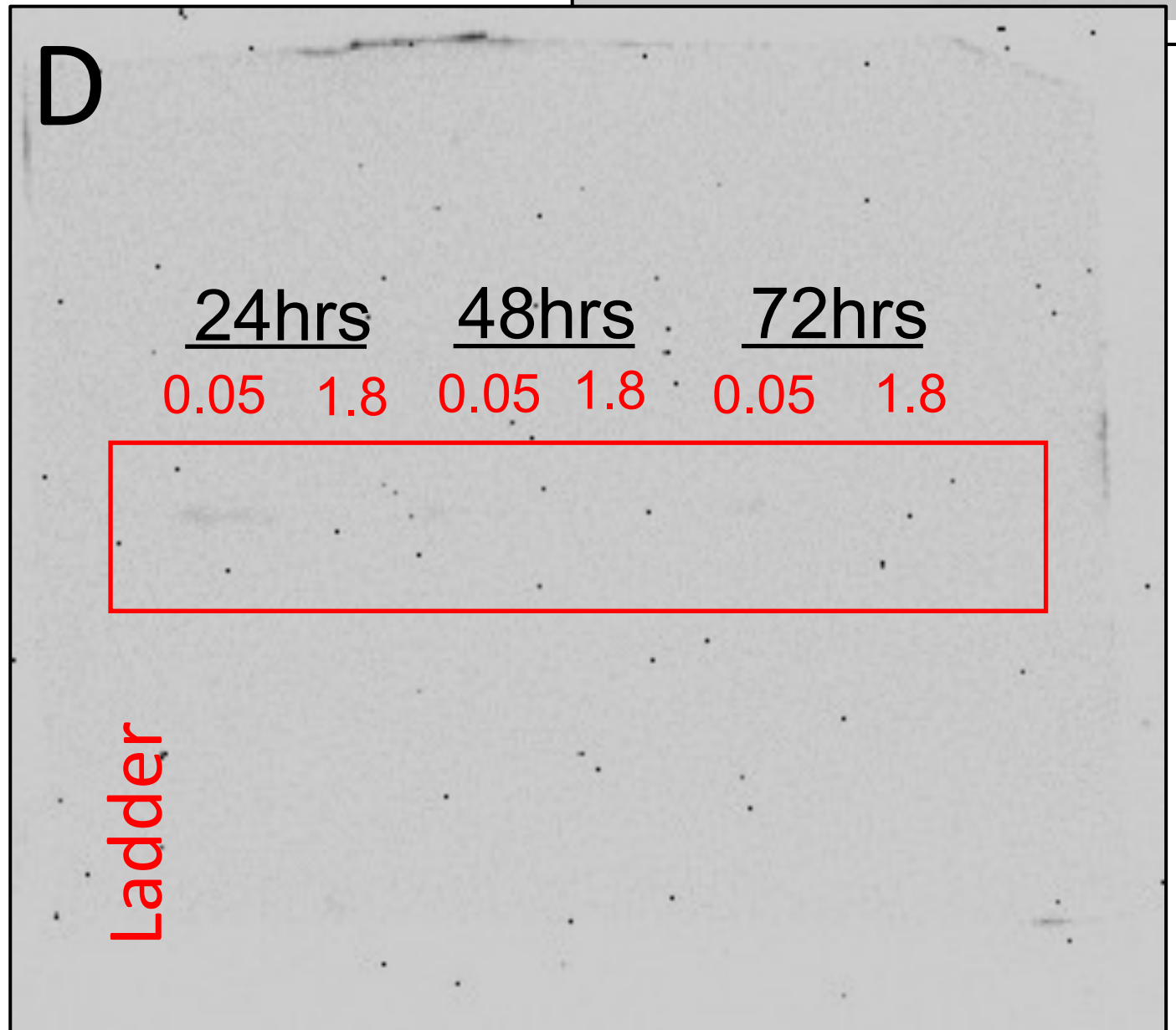

Figure 5e
GATA-3 - 55kDa (Panel A)
GAPDH - 36kDa (Panel B)

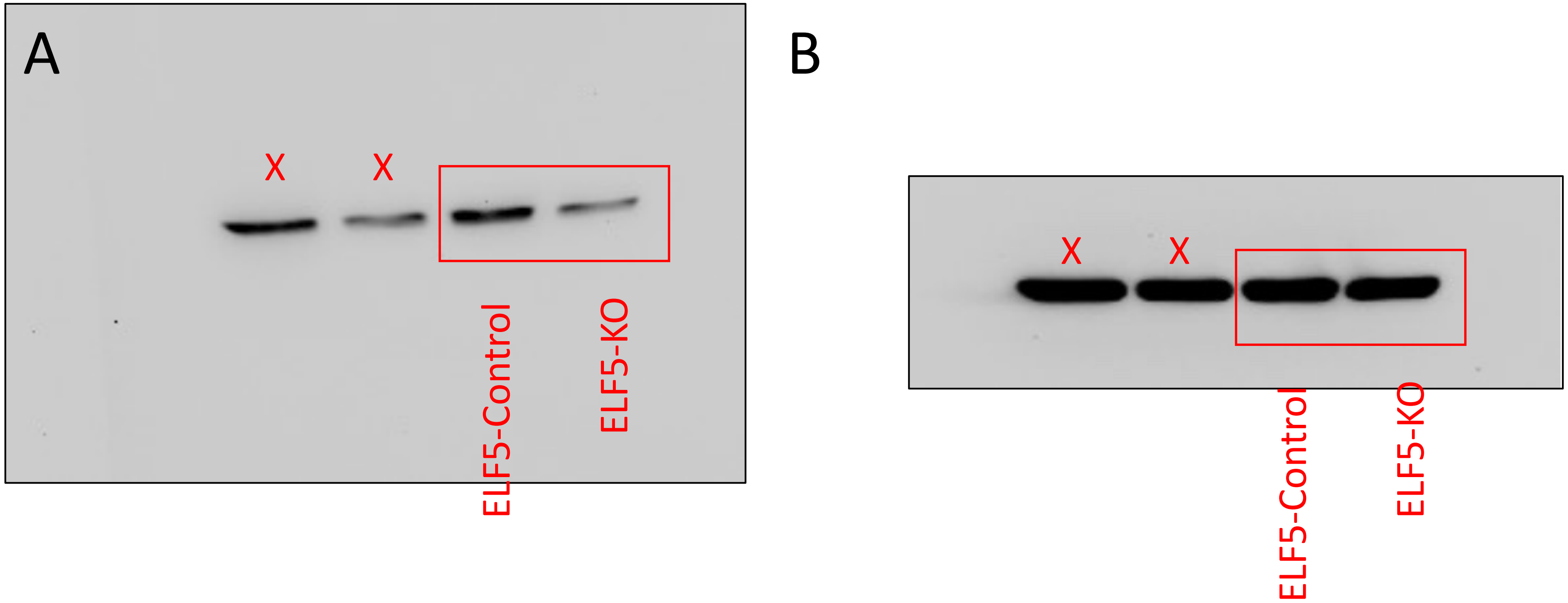

Figure 5e:
FLI-1 - 50kDa (Panel A)
GAPDH - 36kDa (Panel B)

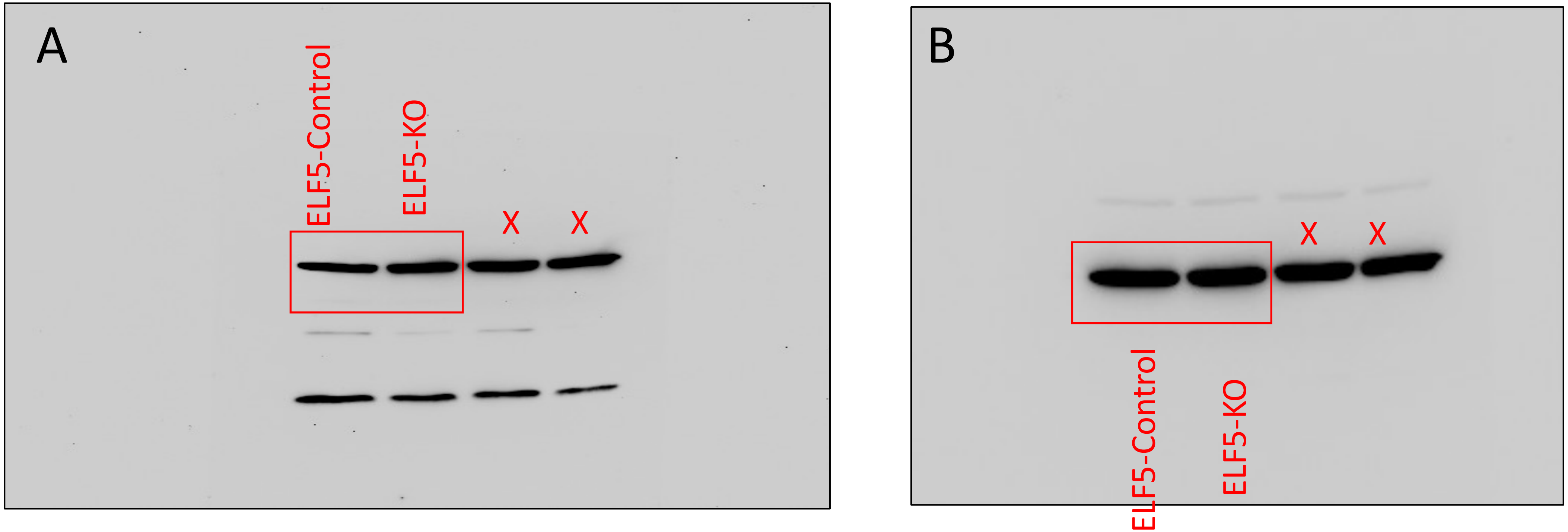

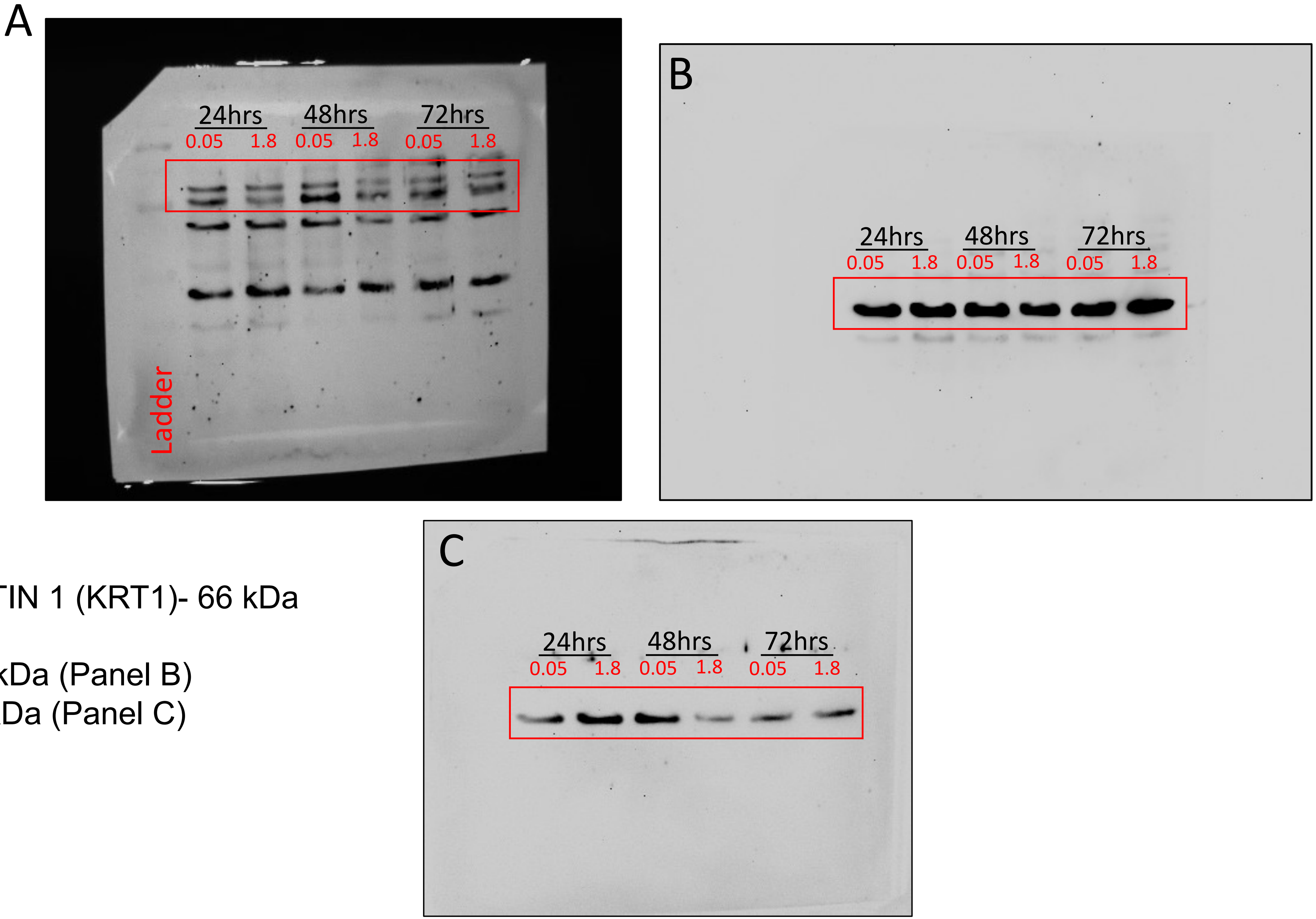

Figure 6B
CYTOKERATIN 1 (KRT1)- 66 kDa (Panel A)
GAPDH - 36kDa (Panel B)
KRT10 – 60kDa (Panel C)

Supplemental Figure S5b:
GATA-3 - 55kDa (Panel A)
GAPDH - 36kDa (Panel B)

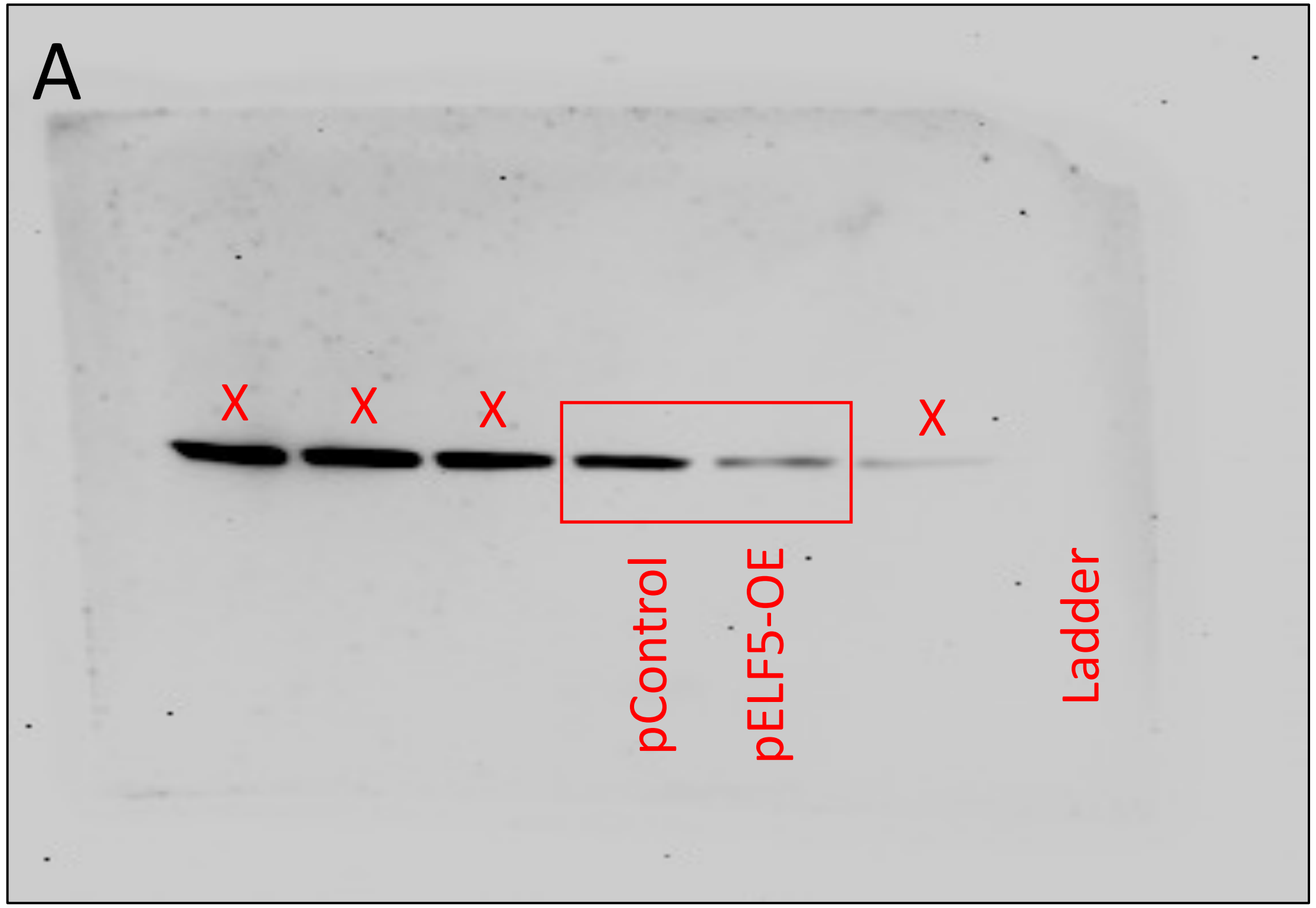

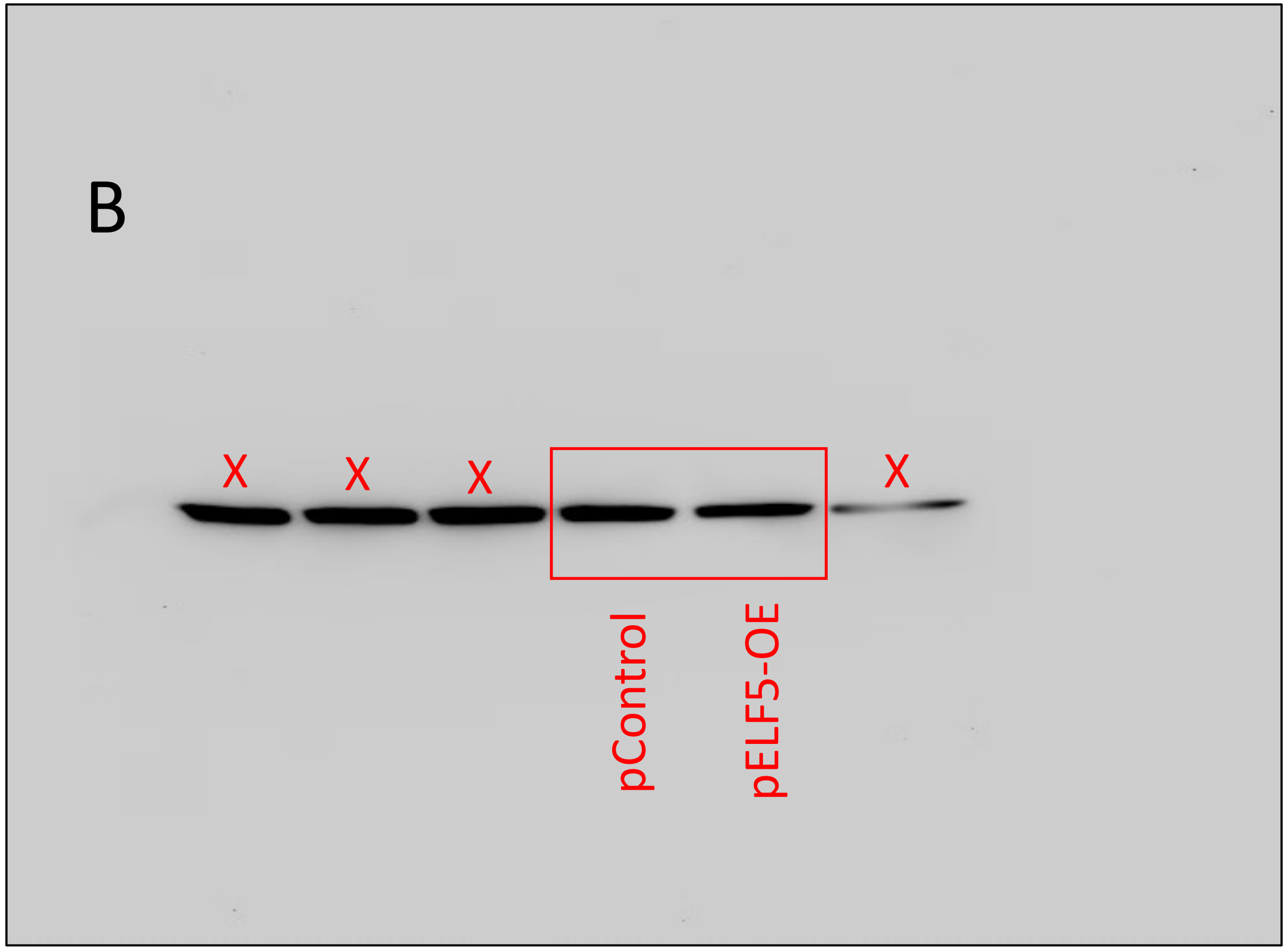

Supplemental Figure S5b:
FLI-1 - 50kDa (Panel A)
GAPDH - 36kDa (Panel B)

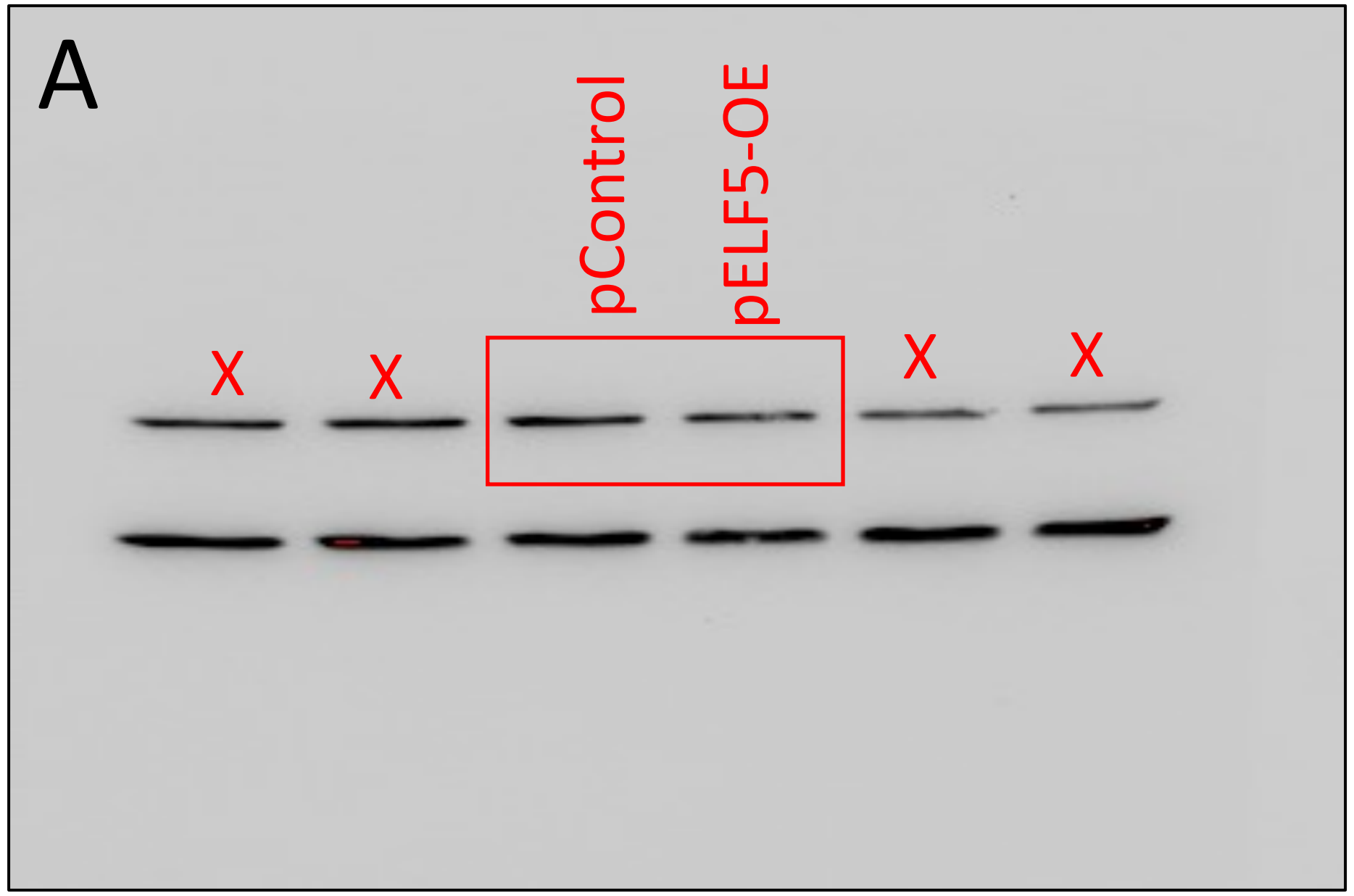

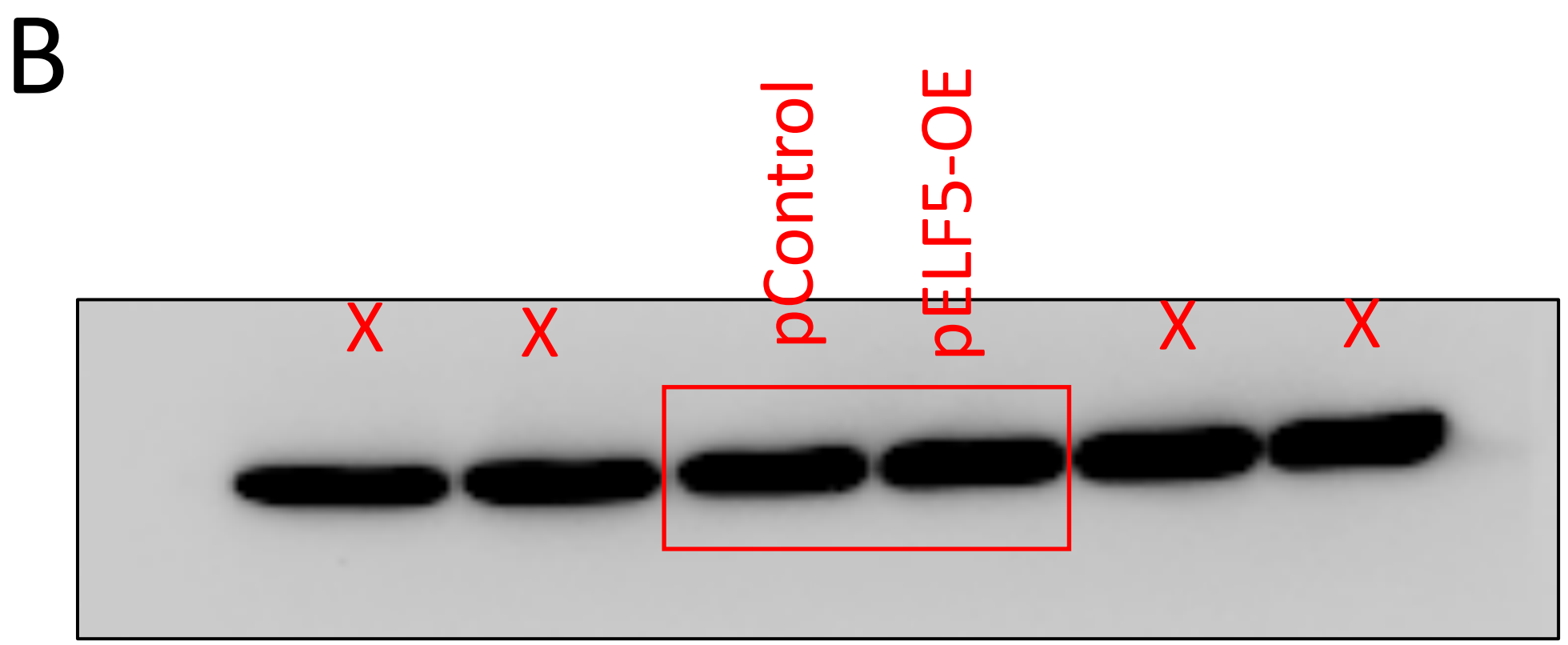

Supplemental Figure S7b:
GATA-3 - 55kDa (Panel A)
GAPDH - 36kDa (Panel B)

A

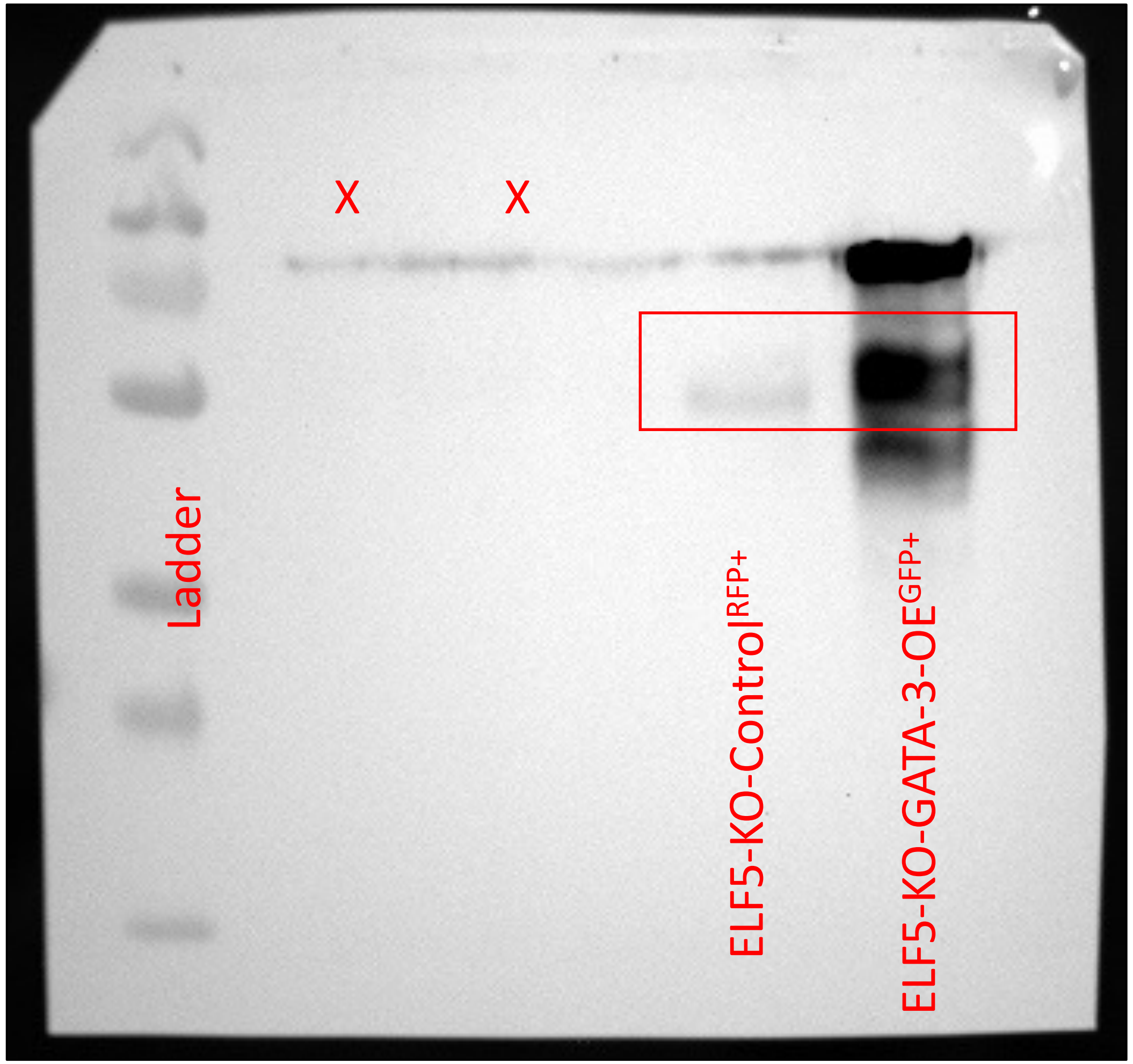

B

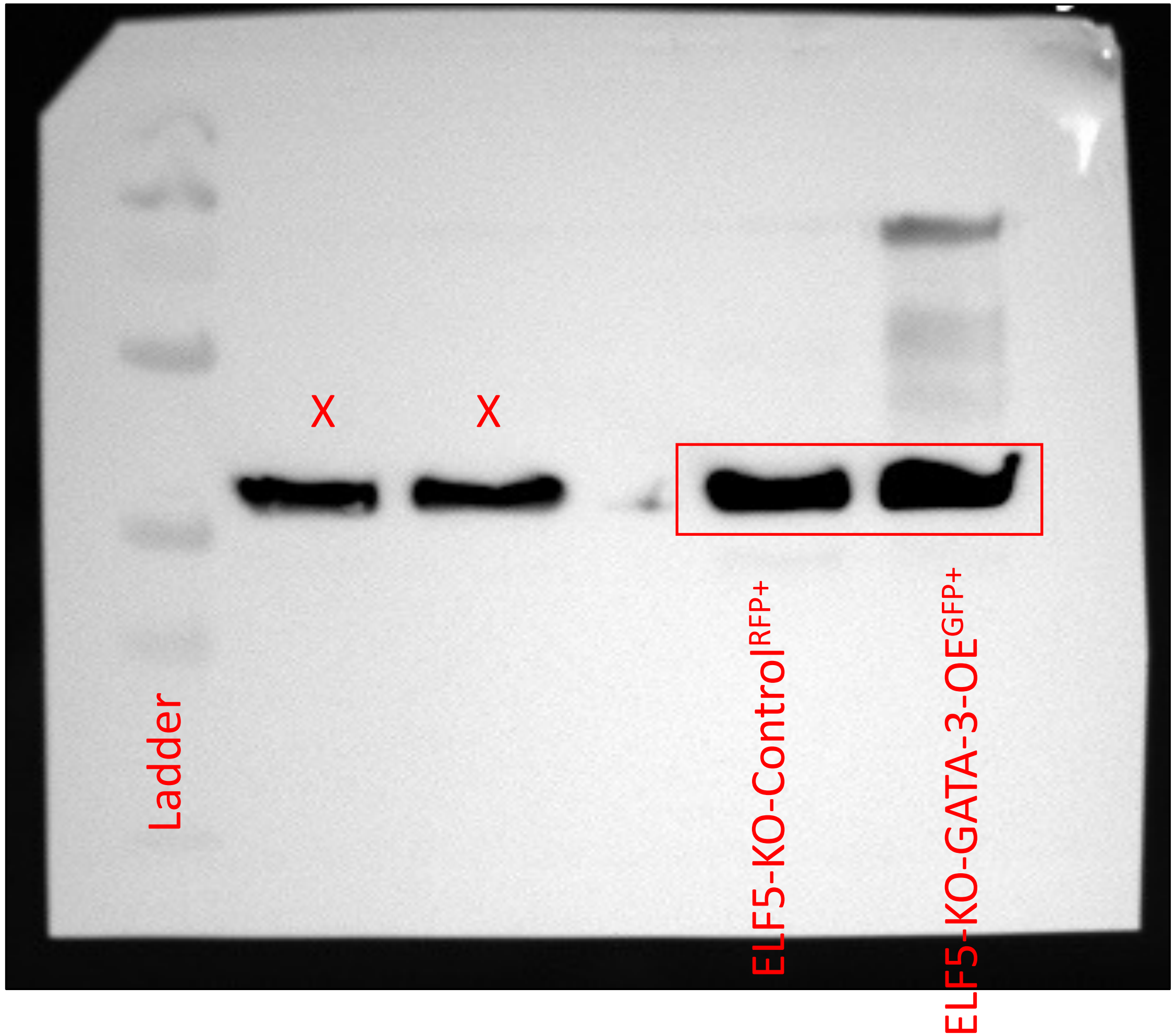

Supplemental Figure S7b:
FLI-1 - 50kDa (Panel A)
GAPDH - 36kDa (Panel B)

A

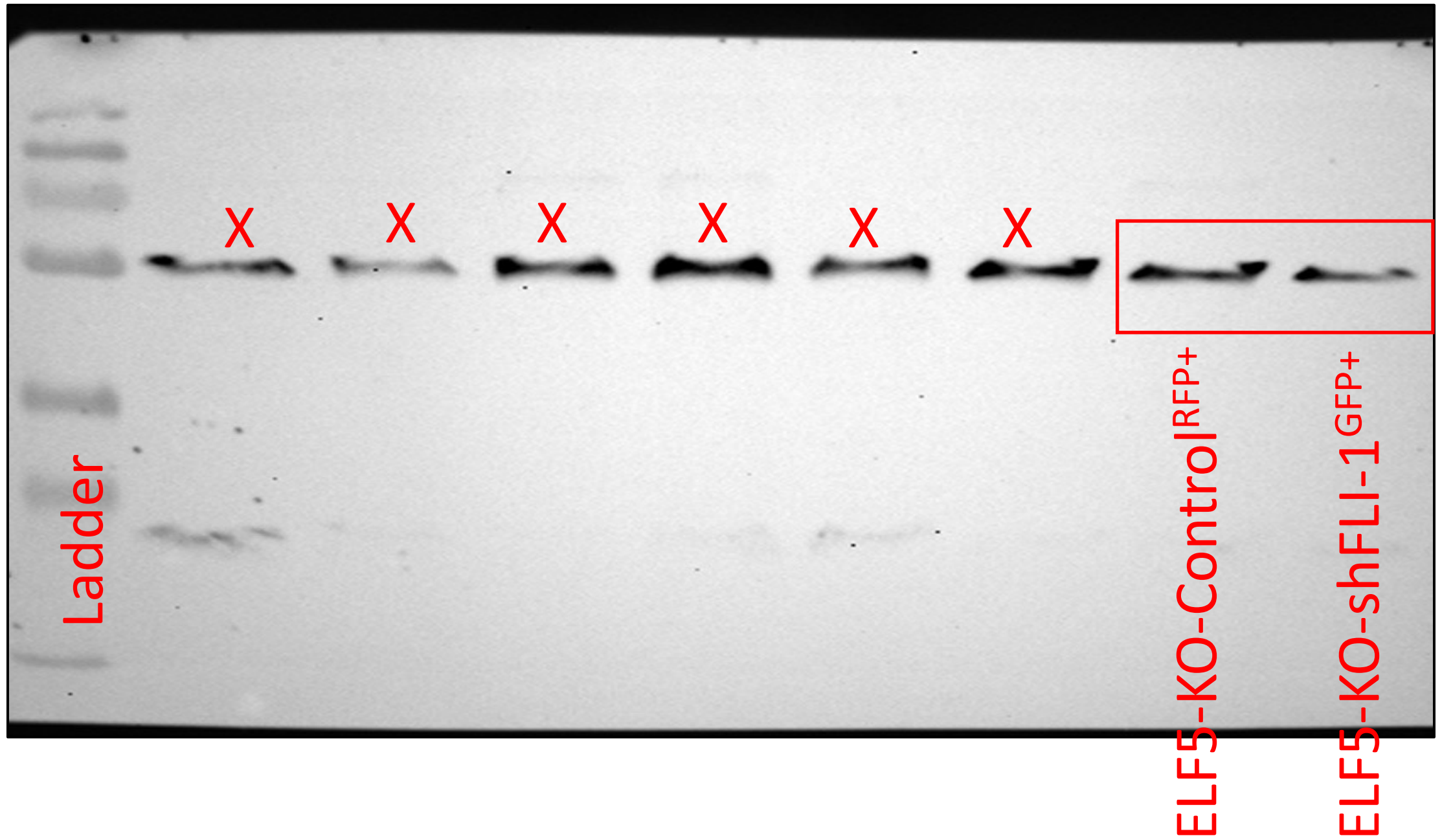

B

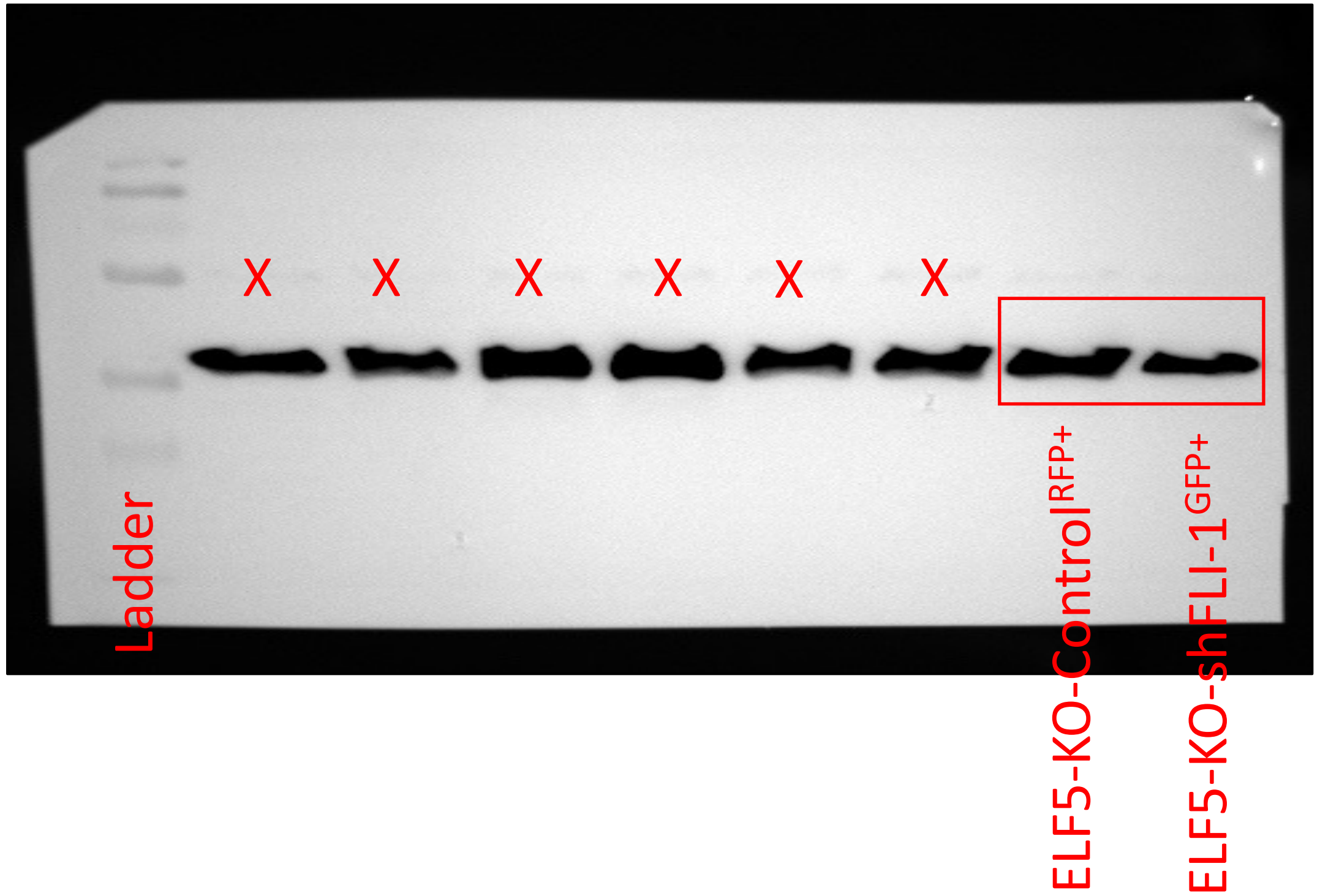

Supplement: Supplementary file 1 [file 41598_2026_59939_MOESM1_ESM.pdf]
