# Supplementary material for: Interplay of ELF5, GATA-3 and FLI-1 regulate malignant transformation of keratinocytes
Source: Sci Rep. 2026 Jul 16;16:25881. doi: 10.1038/s41598-026-59939-0 (PMC13486807; doi:10.1038/s41598-026-59939-0)
Supplement: Supplementary file 2 [file 41598_2026_59939_MOESM3_ESM.pdf]

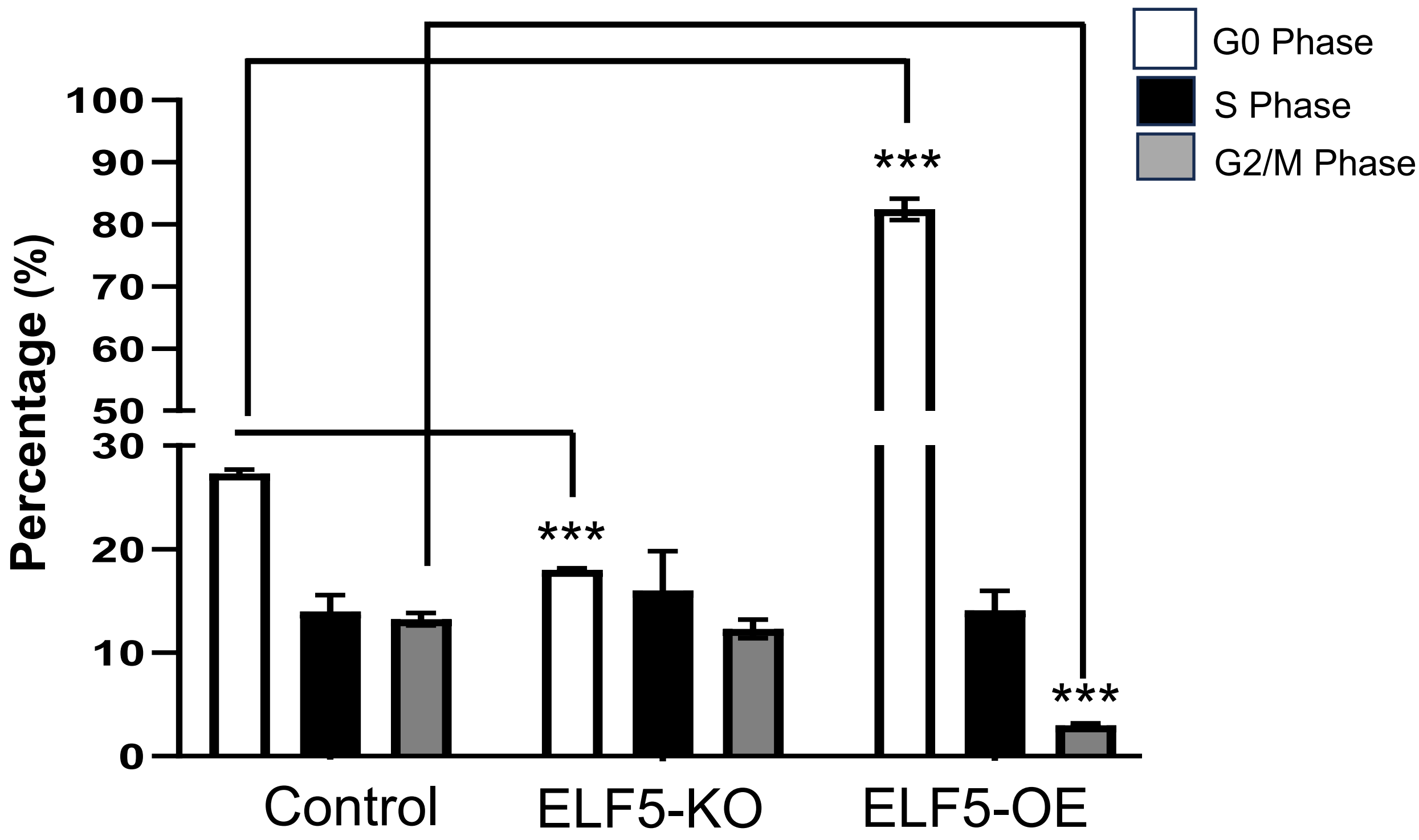

Supplemental Figure S1

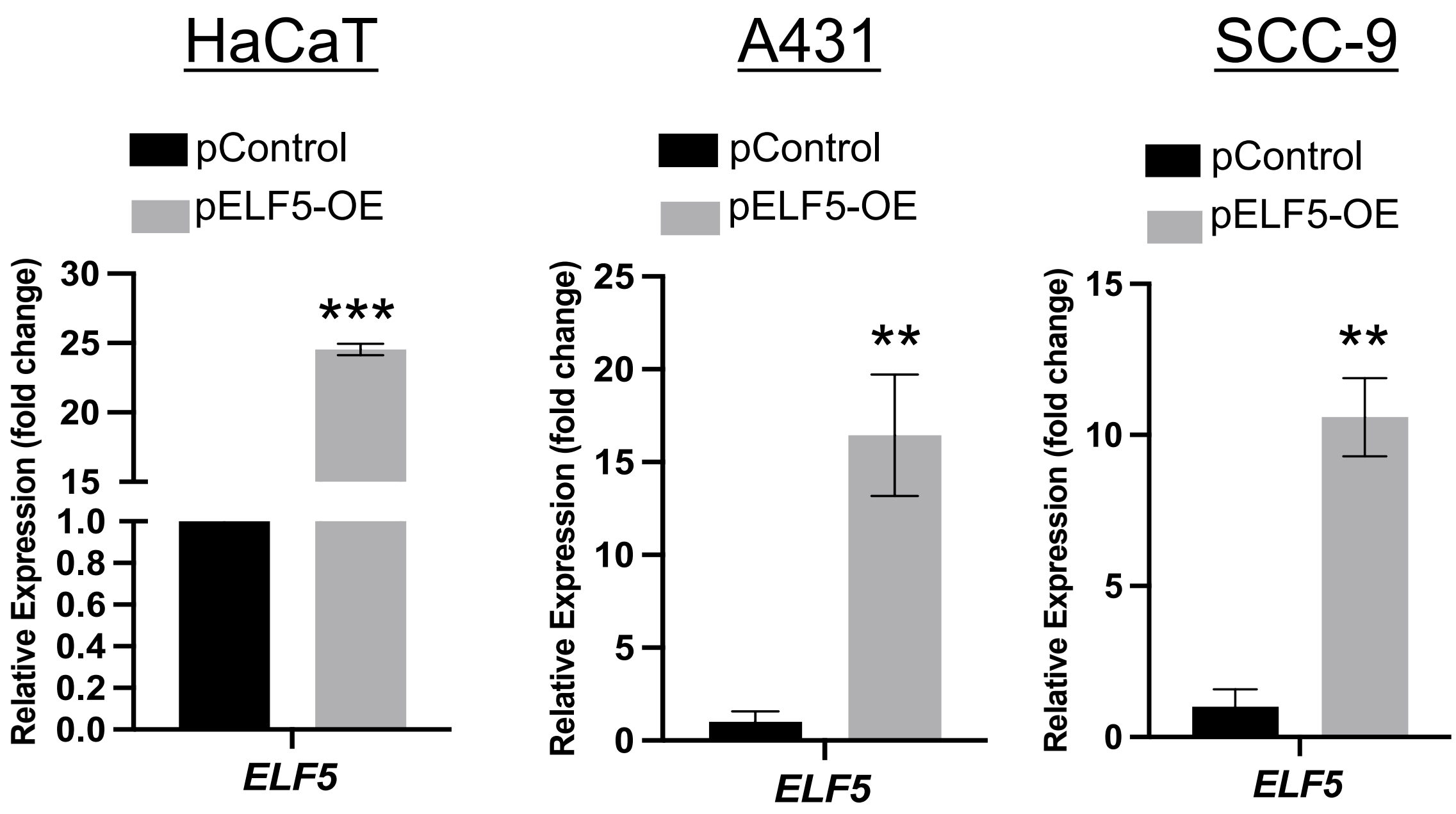

Supplemental Figure S2

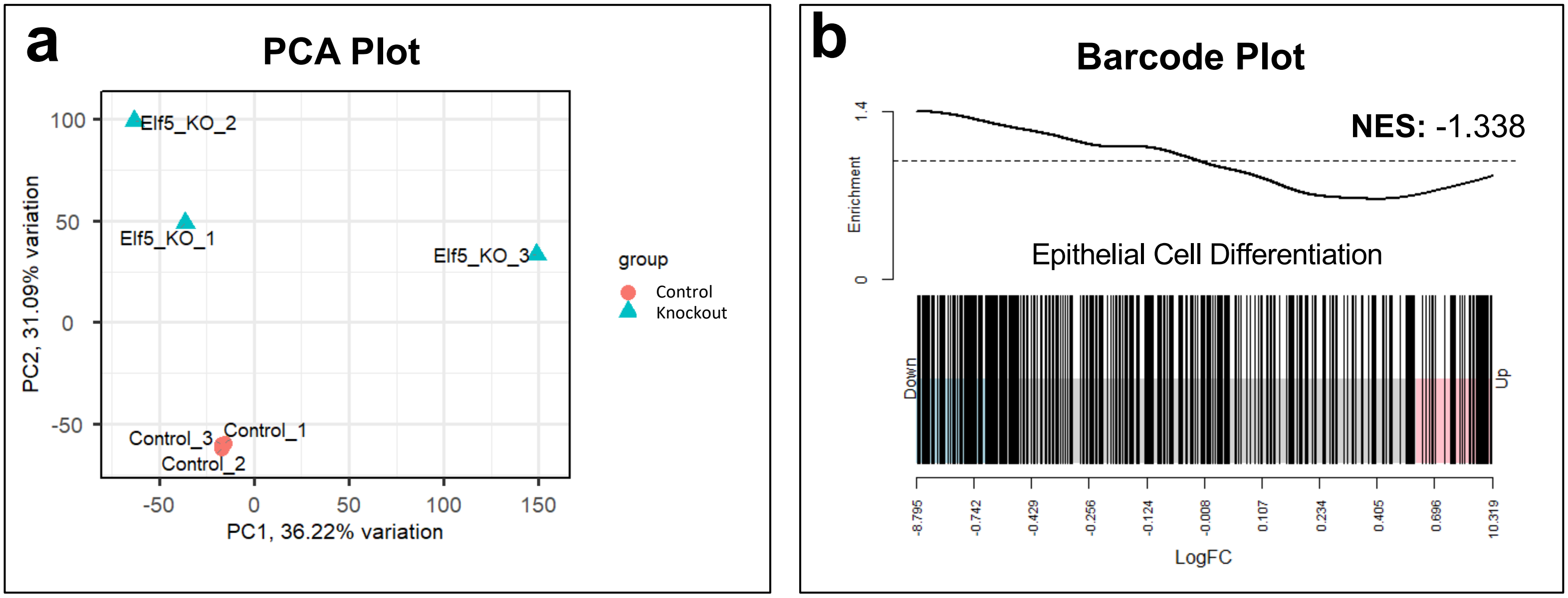

Supplemental Figure S3

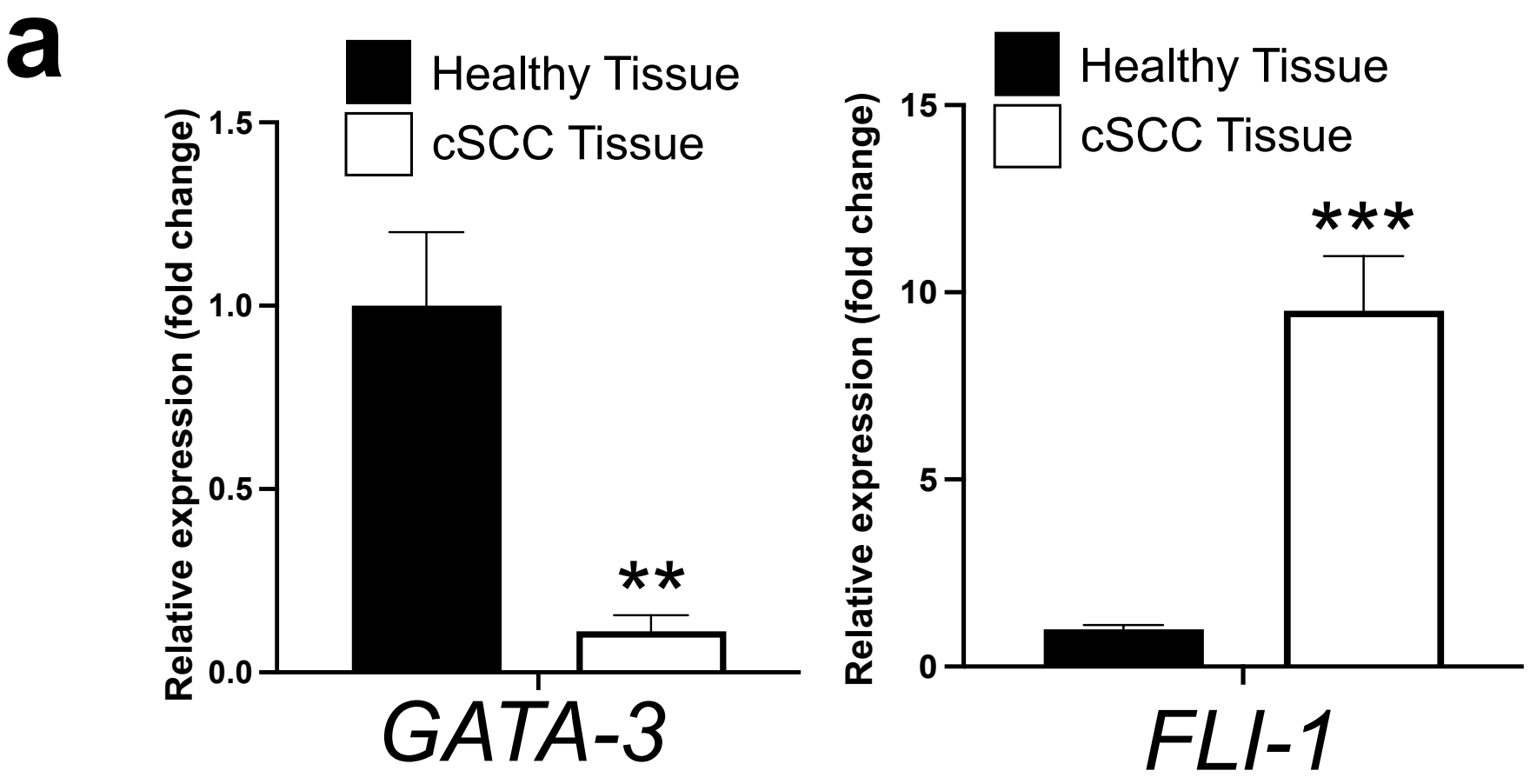

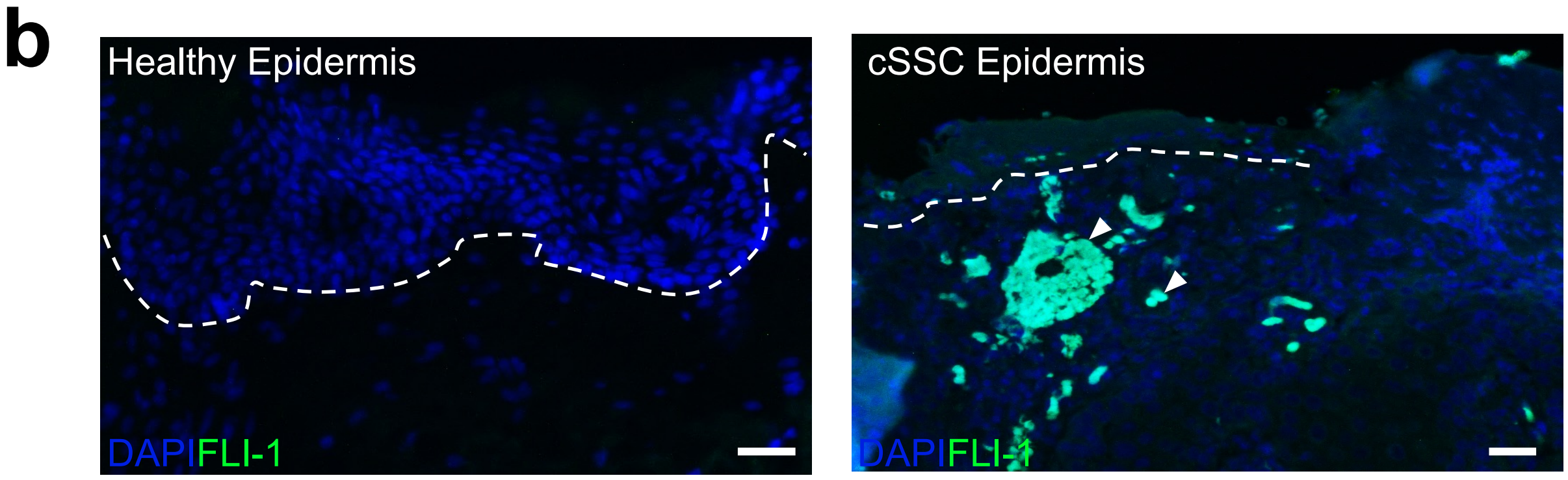

Supplemental Figure S4

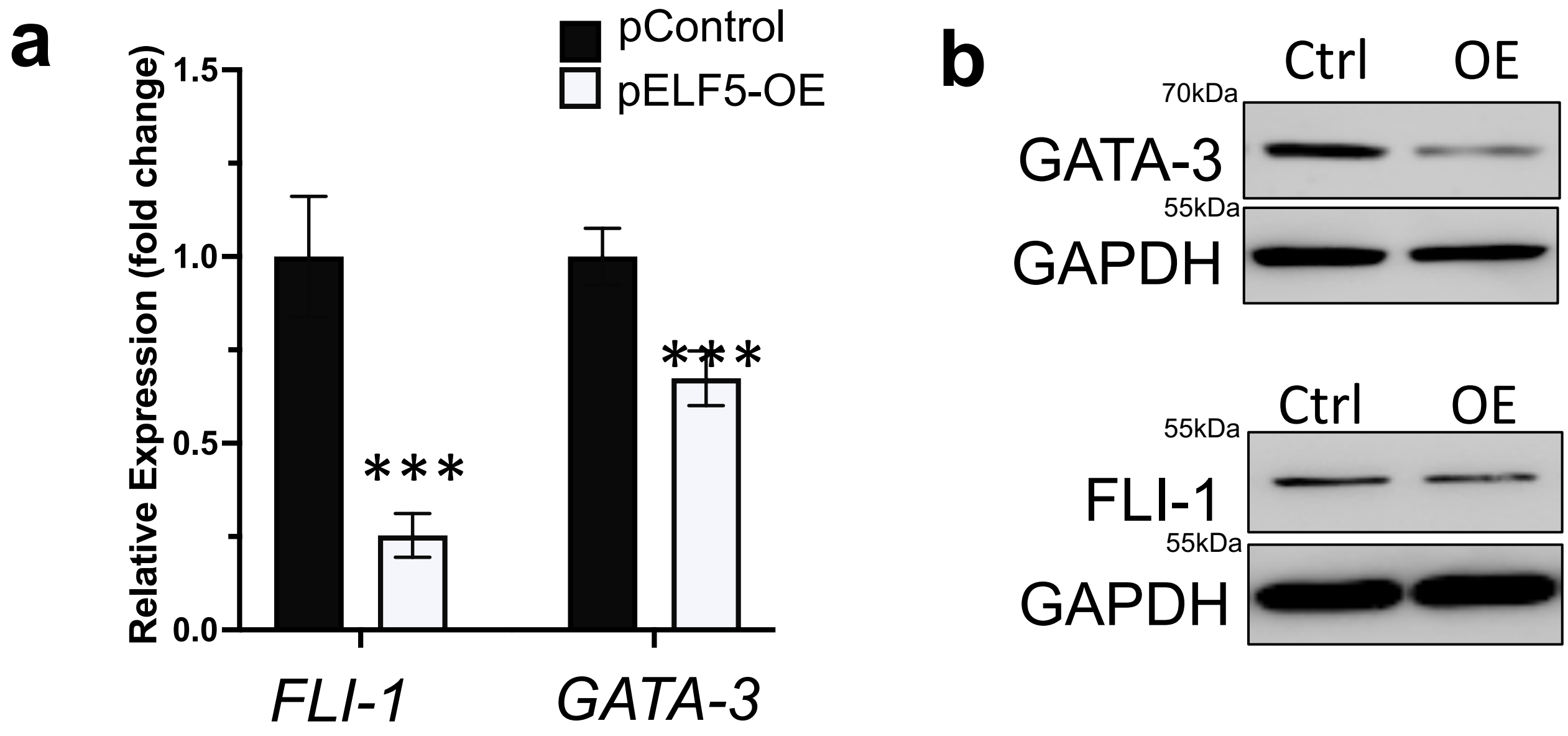

Supplemental Figure S5

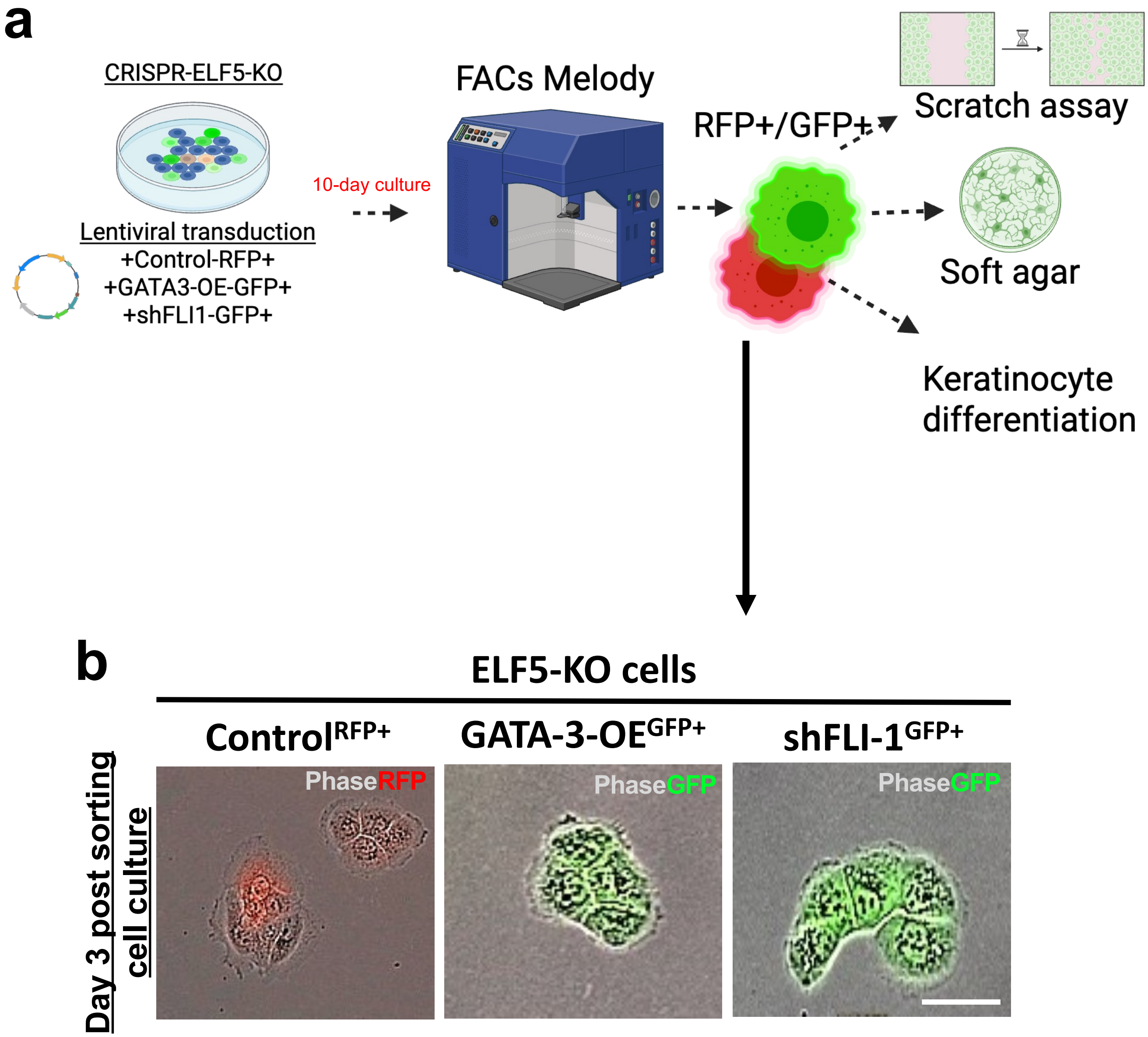

Supplemental Figure S6

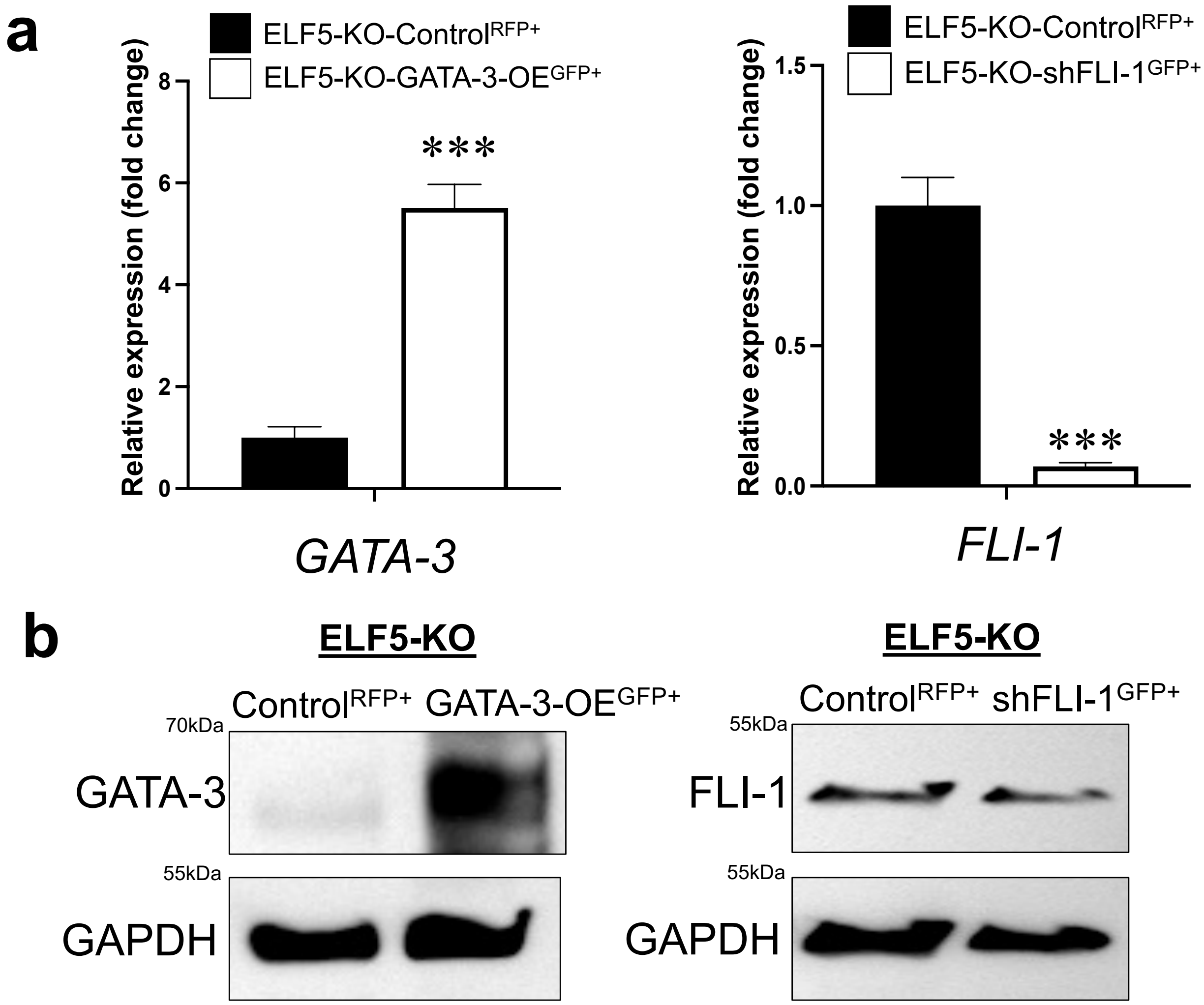

Supplemental Figure S7

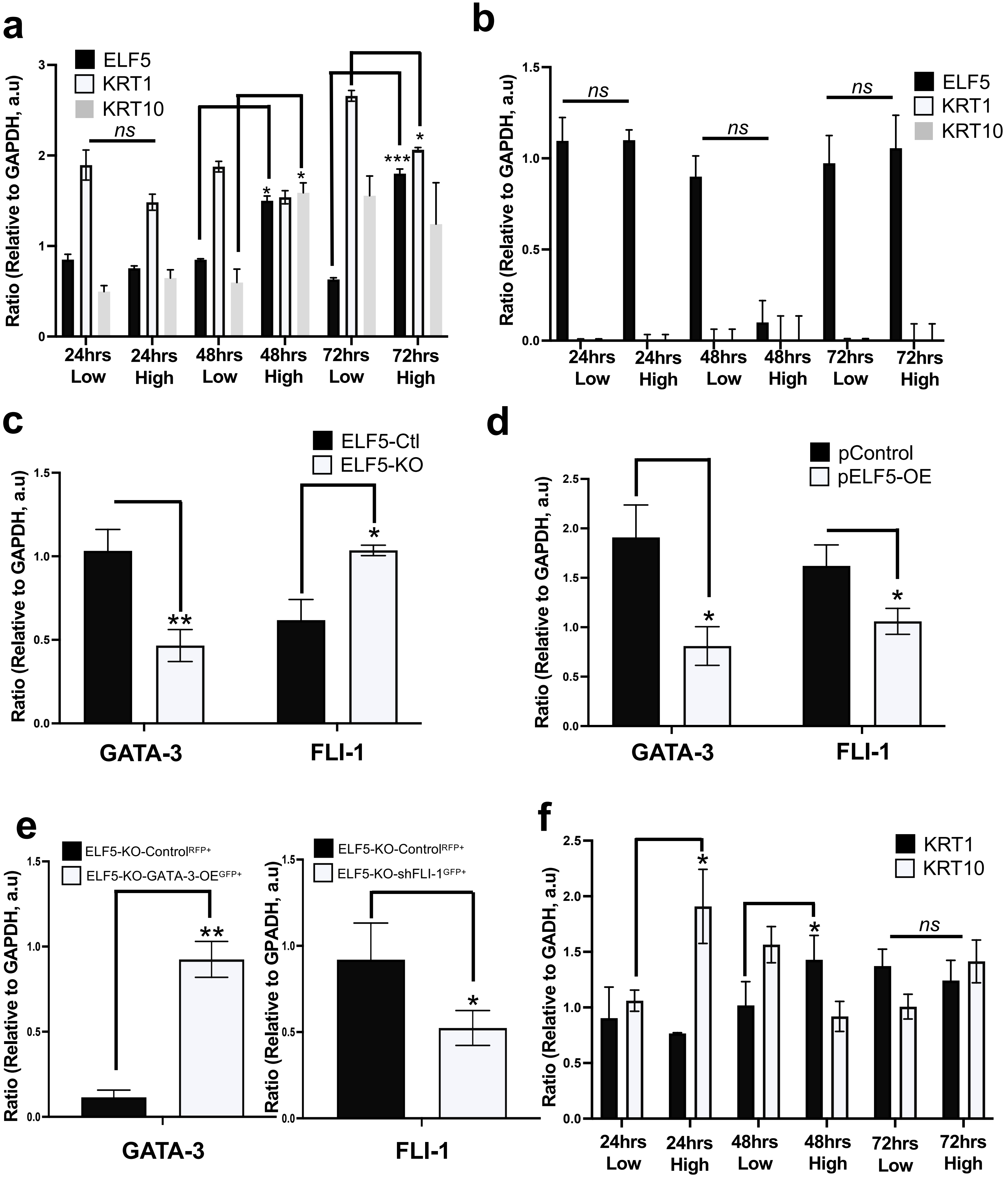

Supplemental Figure S8
