# Supplementary material for: Interplay of ELF5, GATA-3 and FLI-1 regulate malignant transformation of keratinocytes
Source: Sci Rep. 2026 Jul 16;16:25881. doi: 10.1038/s41598-026-59939-0 (PMC13486807; doi:10.1038/s41598-026-59939-0)
Supplement: Supplementary file 3 [file 41598_2026_59939_MOESM2_ESM.docx]

**Supplemental Figure S1.** Statistical analysis of cell cycle analysis. Flow cytometric analysis by Propidium Iodide (PI) revealed that loss of ELF5 (CRISPR/Cas9 HaCaT-ELF5 knockout cells; ELF5-KO) showed significantly reduced cell population in G0 Phase, while cells progressing through the cell cycle were not significantly impacted compared to Control cells. Overexpression of ELF5 (ELF5-OE) leads to significant G0-phase arrest of cells and significantly reduced percentage of cells in G2/M compared to Control cells. The graphs shown are average percentages mean values from three independent experiments. ***p < 0.001, unpaired Student’s *t*-test.

**Supplemental Figure S2.** **Validation of ELF5 overexpression in HaCaT and cancerous cell lines.** RT-qPCR; we confirmed that we were able to successfully transiently overexpress *ELF5* (pELF5-OE) in all cells lines compared to controls cells (pControl). Data are presented as mean ± SEM values from three experimental repeats per group. ***p*<0.01; ****p*<0.001, unpaired Student’s *t*-test.

**Supplemental Figure S3. RNA sequencing analysis**. (**a**) Principal component analysis (PCA) depicting variance between CRISPR/Cas9 HaCaT-ELF5 knockout cells (Knockout, ELF5-KO) *versus* CRISPR/Cas9 HaCaT-ELF5 Control cells (Controls) samples for principal component (PC) 1 and 2. (**b**) A barcode enrichment plot of the significant gene ontology term ‘epithelial cell differentiation’ which is predominantly enriched in downregulated genes after loss of ELF5 with a normalized enrichment score (NES) of -1.337.

**Supplemental Figure S4. GATA-3 and FLI-1 expressional analysis in healthy and cSCC tissues.** (**a**) Using RT-qPCR; we observed that GATA-3 transcript was significantly reduced in cSCC samples compared to healthy tissue. Conversely, FLI-1 expression was significantly increased in transcript and protein levels in cSCC tissue biopsies (arrowheads, tumour cells) compared to healthy skin tissue, which were negative for FLI-1 expression (panel b). DAPI: Blue, FLI-1: Green. Data are presented as mean ± SEM values from three experimental repeats. *n*=3 samples per tissue. Dashed line demarcates epidermal-dermal boarder. ***p*<0.01; ****p*<0.001, unpaired Student’s *t*-test. Scale bar: 50μm

**Supplemental Figure S5. Overexpression of ELF5 leads to downregulation of both GATA-3 and FLI-1 in keratinocytes.** (**a-b**) RT-qPCR and protein analysis; we observed that both GATA-3 and FLI-1 expression was significantly reduced after ELF5 overexpression (pELF5-OE, OE) compared to control cells (pControl, Ctrl) in HaCaT cells. RT-qPCR data are presented as mean ± SEM values from three experimental repeats per group. ****p*<0.001. Western blot data shown are from a single representative experiment from three independent experiments. Densiometric analysis shown in Supplemental Figure S8d.

**Supplemental Figure S6. FACs isolation of ELF5-KO cell variants.** (**a**) CRISPR-Cas9-mediated *ELF5* knockout cells (CRISPR-ELF5-KO) cells were grown and subsequently transduced separately with lentivirals for the following: Control virus (Control^RFP+^), GATA-3 overexpression (GATA-3-OE^GFP+^) and knockdown of FLI-1 (shFLI-1^GFP+^) viruses. These cells were then grown for subsequent experiments post sorting. (**b**) Three days post sorting: cells are still positive for RFP and GFP signals in culture. Image created using Biorender.com.

**Supplemental Figure S7.** **Validation of FACs isolated ELF5-KO cell variants**. (**a-b**) RT-qPCR and protein analysis; we observed that GATA-3 expression was elevated in ELF5-KO-GATA-3-OE^GFP+^ (GATA-3-OE^GFP+^) and FLI-1 expression was significantly decreased in ELF5-KO-shFLI-1^GFP+^ (shFLI-1^GFP+^), compared to ELF5-Control^RFP+^ (Control^RFP+^) cells, respectively. RT-qPCR data are presented as mean ± SEM values from three experimental repeats per group. Western blot data shown are from a single representative experiment from three independent experiments. Densiometric analysis shown in Supplemental Figure S8e. ****p*<0.001, unpaired Student’s *t*-test.

**Supplemental Figure S8. Densiometric analysis.** (**a-f**) Densiometric analysis was performed using Image J (https://imagej.net/ij/). Data shown as ratio relative to GAPDH (arbuitry units, a.u.) with standard error of the mean (± SEM) from three independent experiments. ns: not significant; *p < 0.05; **p < 0.01; ***p < 0.001; unpaired Student’s *t*-test. CYTOKERATIN 1 and 10 (KRT1 and KRT10). Low: low calcium (0.05mM), High: high calcium (1.8mM).
